# Belt electrode tetanus muscle stimulation reduces denervation-induced atrophy of rat multiple skeletal muscle groups

**DOI:** 10.1038/s41598-024-56382-x

**Published:** 2024-03-11

**Authors:** Hiroyuki Uno, Shohei Kamiya, Ryuji Akimoto, Katsu Hosoki, Shunta Tadano, Mako Isemura, Karina Kouzaki, Yuki Tamura, Takaya Kotani, Koichi Nakazato

**Affiliations:** 1 HOMERION LABORATORY Co., Ltd., Shinsen 17-2, Shibuya-Ku, Tokyo, 150-0045 Japan; 2 School of Health and Sport Science, Nippon Sport Science University, 7-1-1 Fukazawa, Setagaya-Ku, Tokyo, 158-8508 Japan

**Keywords:** Biochemistry, Medical research

## Abstract

Belt electrode-skeletal muscle electrical stimulation (B-SES) involves the use of belt-shaped electrodes to contract multiple muscle groups simultaneously. Twitch contractions have been demonstrated to protect against denervation-induced muscle atrophy in rats, possibly through mitochondrial biosynthesis. This study examined whether inducing tetanus contractions with B-SES suppresses muscle atrophy and identified the underlying molecular mechanisms. We evaluated the effects of acute (60 Hz, 5 min) and chronic (60 Hz, 5 min, every alternate day for one week) B-SES on the tibialis anterior (TA) and gastrocnemius (GAS) muscles in Sprague–Dawley rats using belt electrodes attached to both ankle joints. After acute stimulation, a significant decrease in the glycogen content was observed in the left and right TA and GAS, suggesting that B-SES causes simultaneous contractions in multiple muscle groups. B-SES enhanced p70S6K phosphorylation, an indicator of the mechanistic target of rapamycin complex 1 activity. During chronic stimulations, rats were divided into control (CONT), denervation-induced atrophy (DEN), and DEN + electrically stimulated with B-SES (DEN + ES) groups. After seven days of treatment, the wet weight (n = 8–11 for each group) and muscle fiber cross-sectional area (CSA, n = 6 for each group) of the TA and GAS muscles were reduced in the DEN and DEN + ES groups compared with that in the CON group. The DEN + ES group showed significantly higher muscle weight and CSA than those in the DEN group. Although RNA-seq and pathway analysis suggested that mitochondrial biogenesis is a critical event in this phenomenon, mitochondrial content showed no difference. In contrast, ribosomal RNA 28S and 18S (n = 6) levels in the DEN + ES group were higher than those in the DEN group, even though RNA-seq showed that the ribosome biogenesis pathway was reduced by electrical stimulation. The mRNA levels of the muscle proteolytic molecules atrogin-1 and MuRF1 were significantly higher in DEN than those in CONT. However, they were more suppressed in DEN + ES than those in DEN. In conclusion, tetanic electrical stimulation of both ankles using belt electrodes effectively reduced denervation-induced atrophy in multiple muscle groups. Furthermore, ribosomal biosynthesis plays a vital role in this phenomenon.

## Introduction

Decreased activity (e.g., in older adults) leads to skeletal muscle atrophy and muscle weakness, which can interfere with daily life. Preventing skeletal muscle deterioration is essential for increasing healthy life expectancy and maintaining quality of life. Electrical muscle stimulation (EMS) has been reported to induce muscle hypertrophy and inhibit atrophy^1,2^. As demonstrated by animal studies and resistance training in humans, EMS activates protein synthesis signaling, resulting in skeletal muscle hypertrophy^3–5^.

Unlike pad-type electrodes, belt electrode-guided electrical stimulation, also known as belt electrode-skeletal muscle electrical stimulation (B-SES), can simultaneously drive multiple muscle groups in the lower extremities^6^. Clinical studies have reported that B-SES inhibits muscle atrophy and improves motor function in patients by simultaneously activating multiple muscle groups^7–9^. We previously reported that twitch contractions with B-SES in rats suppress denervation-induced muscle atrophy in multiple muscle groups of both legs while maintaining mitochondrial mass and enzyme activity and reducing muscle proteolysis. Pad electrodes with small surface areas fail to produce the same effect observed with belt electrodes using a similar electrical current^6^.

The tetanus EMS with pad-type electrodes effectively mimics resistance exercises and training in animal models^5,10^. The primary mechanism underlying this effect is the activation of mammalian and mechanical targets of rapamycin complex 1 (mTORC1) signaling^11^. p70S6K is a key marker of mTORC1 activation, and p70S6K phosphorylation improves the efficiency of the protein translation machinery in muscles, leading to increased protein synthesis and muscle hypertrophy^4,10^. In addition, ribosomal mass is related to muscle mass and protein synthesis^13,14^. Tetanus EMS in rats increases the number of ribosomes by activating their synthesis^15^. 18S, 28S, and 5.8S rRNAs are cleaved from the rDNA precursor 45S rRNA (45S pre-rRNA)^10,12,16,17^. UBF, TIF-1A, and c-myc regulate rRNA transcription, and the presence of other factors besides mTORC1^10,12,18–20^ suggests that they are regulated by factors other than the mTORC1 pathway^34^. Since skeletal muscle mass is thought to be regulated by the balance between muscle protein synthesis and degradation^21–23^, we hypothesized that tetanic contractions induced by B-SES would suppress muscle atrophy^6^.

In this study, we used a previously developed rodent B-SES model^6^ to test whether the electrical stimulation of two ankle belt electrodes in both legs can activate multiple muscle groups. We first observed significant glycogen consumption and p70S6K phosphorylation by high-frequency stimulation-induced tetanic contractions to confirm the activation of multiple skeletal muscles by belt electrodes. In one week of chronic stimulation, we observed that tetanic-contraction treatment reduced the degree of denervation-induced muscle atrophy in multiple skeletal muscles, as previously observed using twitch contractions^6^. We further performed RNA-seq to gain a holistic picture of this phenomenon. We examined mitochondrial and ribosomal biogenesis, content, and muscle proteolytic signaling to elucidate the underlying molecular mechanisms.

## Result

We previously reported that twitch B-SES induces contractions in multiple lower extremity muscle groups^6^. In addition, p70S6K phosphorylation is a key marker of mTORC1 signaling activation. p70S6K phosphorylation increases muscle protein translation efficiency and mitochondrial biosynthesis, leading to protein synthesis and muscle hypertrophy^4,10,11^. Therefore, we evaluated whether tetanus B-SES activates multiple muscles in the lower extremities and promotes muscle protein synthesis by quantifying glycogen levels and p70S6K.

**Figure 1:**
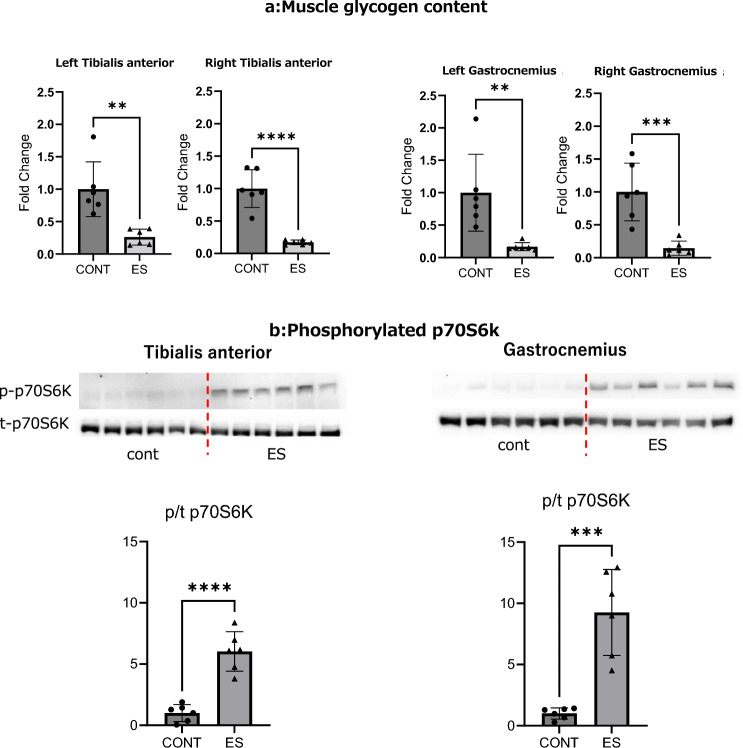
Glycogen content of TA and GAS and phosphorylated p70S6K after acute belt electrode stimulation (mean ± SD). The unpaired t-test was used to evaluate changes in (**a**) glycogen content and (**b**) phosphorylated p70S6K of leg muscles immediately after acute stimulation by the belt electrode. CONT: no stimulation control group, ES: Electrostimulation with belt electrode group, *p < 0.05, **p < 0.01, ***p < 0.001, ****p < 0.0001.

Immediately after stimulation, the glycogen levels decreased in the tibialis anterior (TA) and gastrocnemius (GAS) muscles of both lower extremities (Fig. 1a). Six hours after acute stimulation, p70S6K phosphorylation was increased in the TA and GAS of both lower limbs (Fig. 1b). These results suggest that B-SES simultaneously activates the anterior and posterior muscles in both lower extremities and promotes muscle protein synthesis.

### Chronic belt electrode skeletal muscle electrical stimulation inhibits denervation-induced muscle atrophy

In chronic stimulation, the effects of tetanus with B-SES on sciatic nerve denervation-induced muscle atrophy were evaluated based on muscle weight and cross-sectional area (CSA).

**Figure 2:**
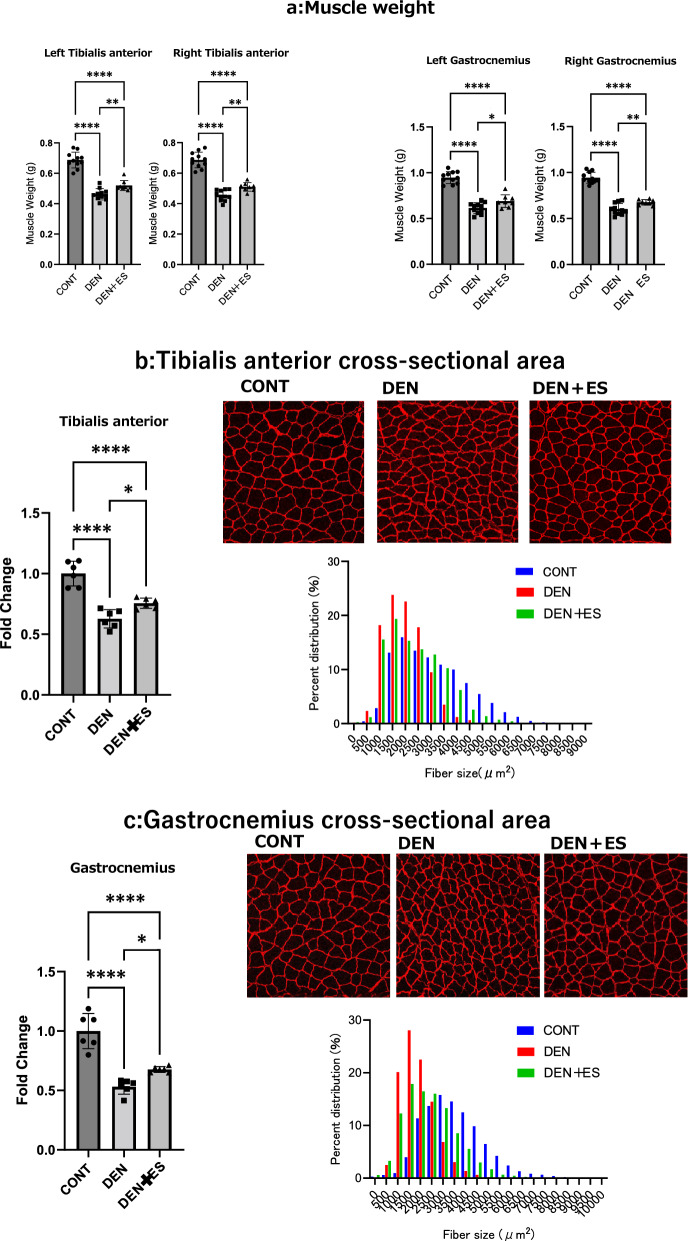
Muscle wet weight and muscle fiber cross-sectional area (CSA) of left and right TA and GAS after chronic belt electrode stimulation (mean ± SD). Changes in muscle wet weight and muscle fiber CSA of lower limb muscles 24 h after chronic stimulation with a belt electrode, as evaluated by one-way analysis of variance; CONT, non-stimulated control group; DEN: denervation muscle atrophy induced group; DEN + ES: denervation muscle atrophy induced + belt electrode electrical stimulation group; *p < 0. 05, **p < 0.01, ***p < 0.001, ****p < 0.0001.

The muscle weight and CSA of the TA and GAS of the denervation-induced muscle atrophy (DEN) and DEN + electrical stimulation (ES) groups were significantly lower compared to those in the control group (CONT); those of the DEN + ES group were significantly higher compared to those in the DEN group (Fig. 2). (Body weight, CONT: 399 ± 17 g, DEN: 373 ± 23 g, DEN + ES: 386 ± 7 g, mean ± SD). The effect sizes shown in Table 1 for the DEN group versus the DEN + ES group were greater than 0.6, suggesting that the difference had sufficient statistical power.

**Table 1:** Effect size of muscle weight (DEN vs DEN + belt ES).

Muscle	Effect size(r)
Left GAS	0.642
Right GAS	0.795
Left TA	0.795
Right TA	0.768

These results suggest that tetanus with B-SES simultaneously inhibits atrophy of multiple muscle groups.

### Gene expression profiling using RNA-Seq

We investigated the molecular mechanism since the tetanic electrical stimulation reduced muscle atrophy. We first performed RNA-seq to investigate the mRNAs and pathways responsible for this phenomenon to gain a complete picture.

**Figure 3:**
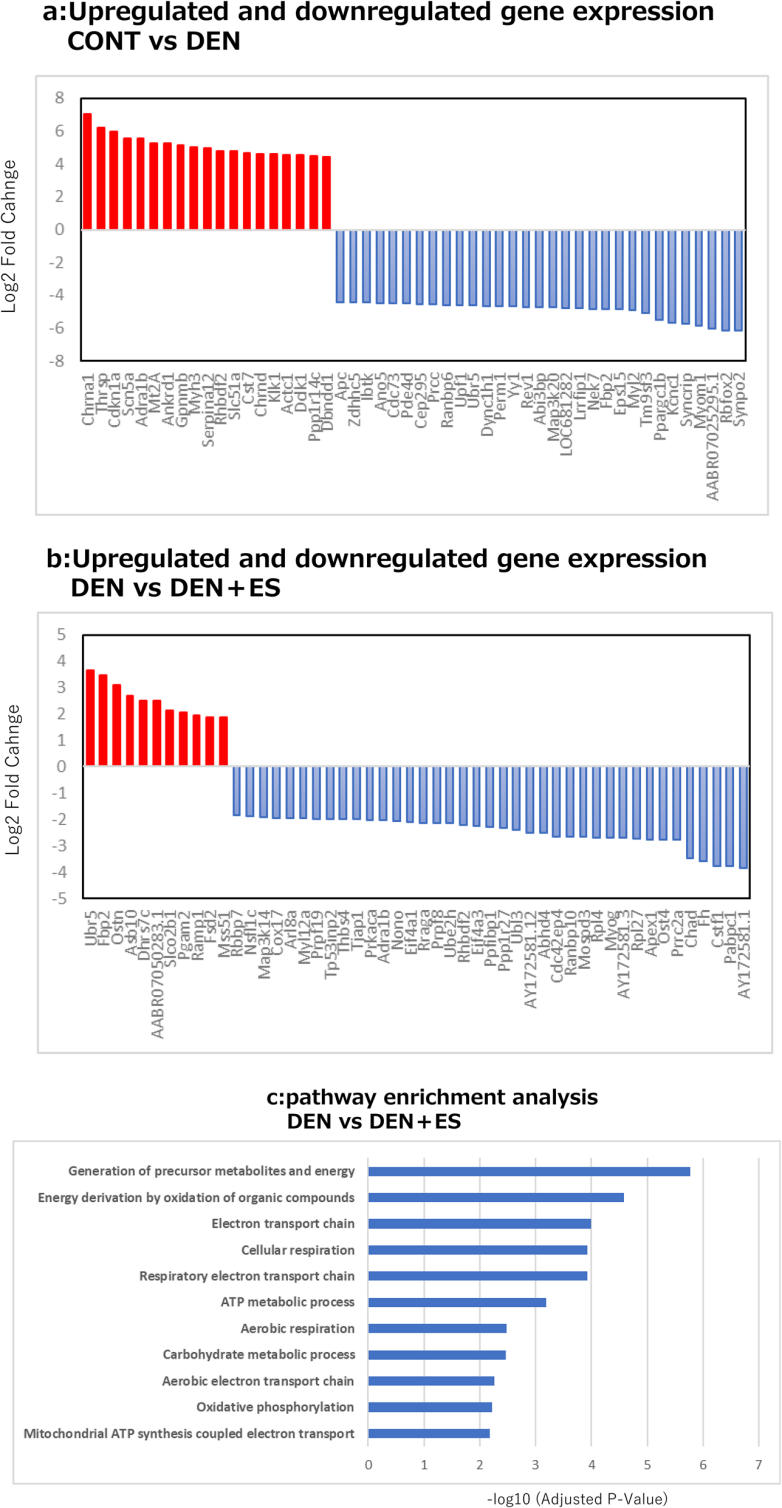
RNA-seq variation gene and pathway analysis after chronic stimulation. RNA-seq analysis of up-and downregulated genes (TOP50) and pathway analysis of genes(c) with altered expression in CONT-DEN (**a**) and DEN-DEN + ES (**b**). CONT: non-stimulated control group, DEN: denervated muscle atrophy induced group, DEN + ES: denervated muscle atrophy induction + electrical stimulation with belt electrode group.

Differences among the CONT, DEN, and DEN + ES groups were evaluated by isolating mRNA one week after the treatment period, and changes in RNA-seq-based gene expression were analyzed. The top 50 genes showing increased or decreased expression were illustrated by significant changes in gene expression observed in mitochondria-related genes (AY172581.1, Fh, Cox17), mTORC signaling, muscle protein synthesis by ribosomes and others (Rpl27, Rpl4, Myog, Eif4a3, Rraga), and muscle protein degradation (Ubr5, Ube2h) (Fig. 3a,b). Pathway analysis showed that mitochondria-related pathways are activated by electrical stimulation (Fig. 3c). In contrast, ribosome-associated mRNAs such as Rpl27 and Rpl4 were decreased by electrical stimulation.

### Effects of chronic belt electrode skeletal muscle electrical stimulation on mitochondrial synthesis and quantity

Since we observed significant activation of mitochondrial biogenesis, we evaluated PGC-1α and COX IV, the primary regulators of mitochondrial synthesis and indicators of mitochondrial mass.

**Figure 4:**
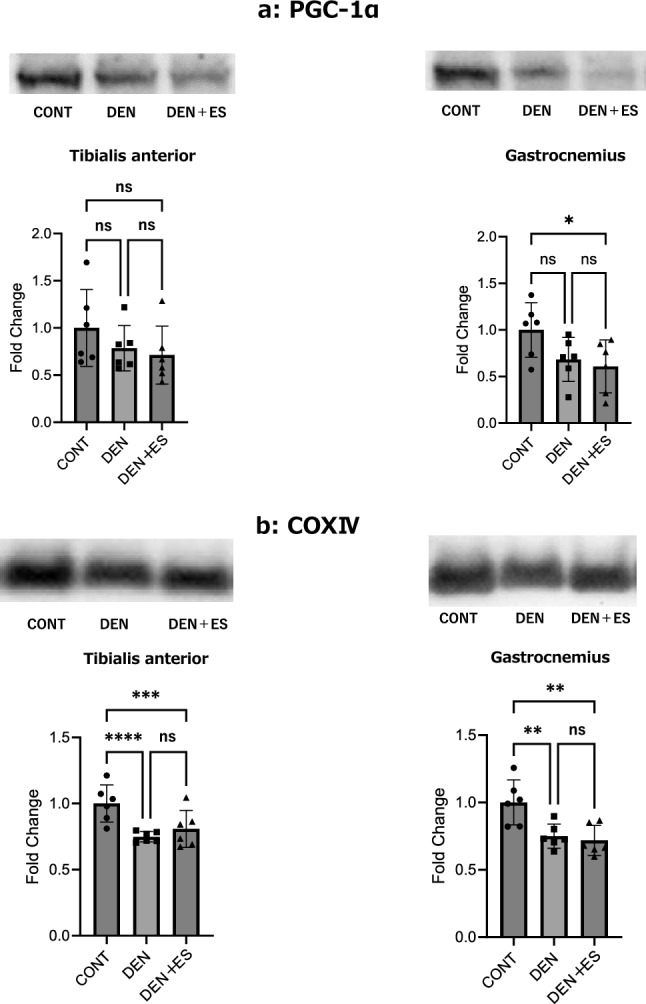
Changes in TA and GAS after chronic belt electrode stimulation (Mean ± SD) in PGC-1α and COXIV levels. (**a**) PGC-1α and (**b**) COXIV levels 24 h after chronic stimulation with a belt electrode were evaluated by one-way analysis of variance. CONT: non-stimulated control group, DEN: denervation muscle atrophy induced group, DEN + ES: denervation muscle atrophy induced + electrical stimulation with belt electrode group, *p < 0.05, **p < 0.01.

The expression of PGC-1α protein decreased in the DEN group compared to that in the CONT group (no significant difference). However, there was no difference between the DEN and DEN + ES groups (Fig. 4a). In addition, COXIV protein levels (Fig. 4b) were significantly decreased in both the TA and GAS in the DEN and DEN + ES groups compared to those in the CONT group; however, there was no significant difference between the DEN and DEN + ES groups, suggesting that B-SES tetanus stimulation did not prevent the denervation-induced decrease in mitochondrial content.

### Ribosomal content after chronic belt electrode skeletal muscle electrical stimulation

Although RNA-seq suggested that mRNAs related to ribosome biogenesis were decreased, ribosomes are regarded as critical factors for muscle protein synthesis, especially after tetanic muscle contractions. Thus, we evaluated the ribosome contents after chronic stimulation.

**Figure 5:**
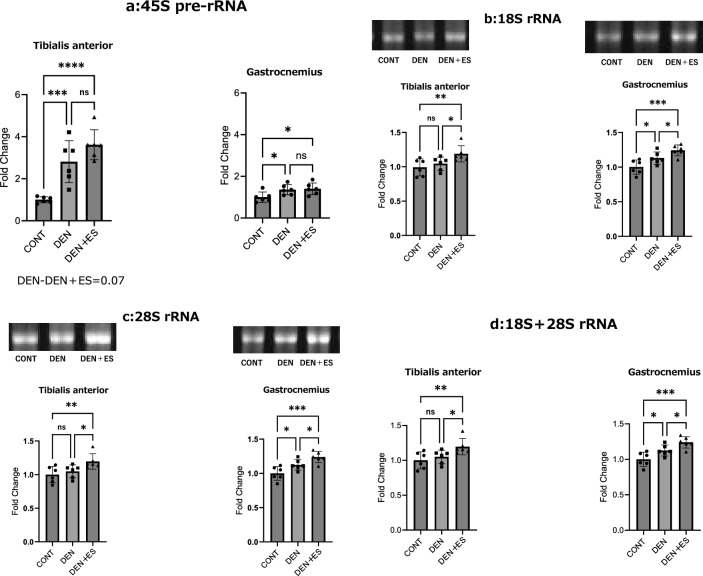
UBF, TIF-1a, and c-myc in TA and GAS after belt electrode chronic stimulation (mean ± SD); changes in (**a**) UBF, (**b**) TIF-1a, and (**c**) c-myc levels in TA and GAS 24 h after chronic stimulation with a belt electrode were evaluated by one-way analysis of variance; CONT: non-stimulated control group, DEN: denervated muscle atrophy induced group, DEN + ES: denervated muscle atrophy induced + belt electrode electrical stimulation group, *p < 0.05.

After chronic stimulation, 18S, 28S rRNA, and 45S pre-rRNA were evaluated using electrophoresis and RT-PCR. 45S rRNA expression showed an increasing trend in TA in the DEN + ES group compared to that in the DEN group; however, there was no significant difference in GAS (Fig. 5a). The 18S and 28S rRNA levels were significantly increased in the DEN + ES group compared to those in the CONT and DEN groups in TA; in GAS, significant increases were observed in the DEN and DEN + ES groups compared to those in the CONT group and the DEN + ES group compared to those in the DEN group (Fig. 5b,c). Similarly, 18S + 28S rRNA levels were significantly increased in the DEN + ES group compared to those in the DEN group for both TA and GAS (Fig. 5d). These results suggest that B-SES tetanus stimulation increases ribosomal content in both the TA and GAS muscles, even though the RNA-seq suggested that electrical stimulation might reduce ribosome biogenesis.

### Ribosome biogenesis by chronic belt electrode skeletal muscle electrical stimulation

**Figure 6:**
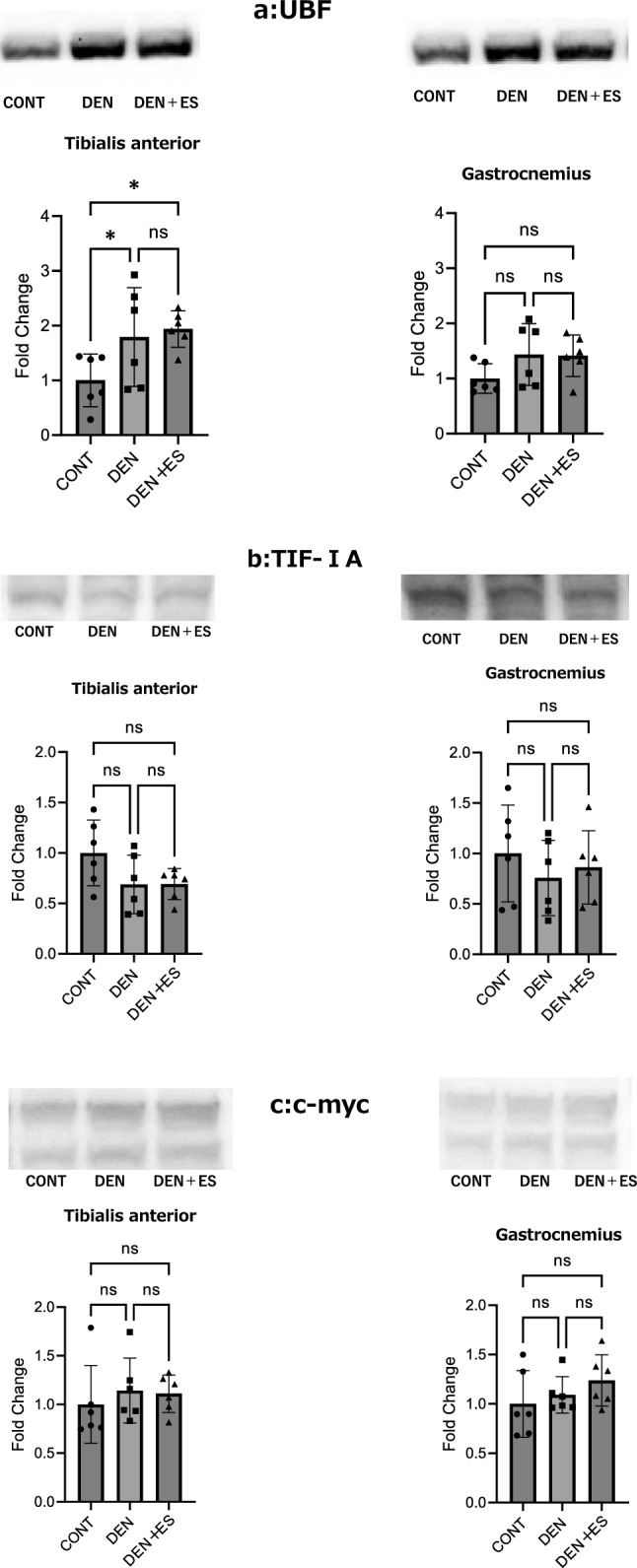
The rRNA levels in the TA and GAS after chronic belt electrode stimulation (mean ± SD) (**a**) 28S, (**b**) 18S, and (**c**) 45S. Changes in 28S, 18S, and 45 rRNA levels of TA and GAS 24 h after chronic stimulation with a belt electrode were evaluated by one-way analysis of variance. CONT: non-stimulated control group, DEN: denervation muscle atrophy induced group, DEN + ES: denervation muscle atrophy induced + belt electrode electrical stimulation group, *p < 0.05, **p < 0.01, ***p < 0.001, ****p < 0.0001.

Next, the expression levels of UBF, TIF-1A, and c-myc, which are involved in ribosome biosynthesis after chronic stimulation, were evaluated using western blotting. At the TA, UBF levels were enhanced in the DEN and DEN + ES groups compared to the CONT group. However, there was no significant difference between the DEN and DEN + ES groups. No significant differences were observed in the GAS muscles among the three groups. TIF-1a and c-myc levels were not significantly different across the three groups, and the effects of denervation and electrical stimulation could not be verified (Fig. 6). The ribosome biogenesis results were essentially the same as those obtained using RNA-seq.

### Muscle proteolytic signals after chronic belt electrode skeletal muscle electrical stimulation

**Figure 7:**
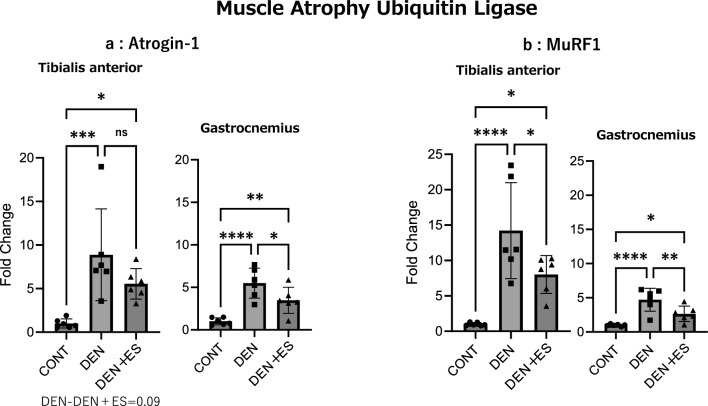
Muscle proteolytic signals of TA and GAS after chronic belt electrode stimulation (mean ± SD). Changes in proteolytic signals of lower limb muscles 24 h after chronic belt electrode stimulation were evaluated by one-way analysis of variance test; CONT: non-stimulated control group; DEN: denervation muscle atrophy induced group; DEN + ES: denervation muscle atrophy induced + electrical stimulation with belt electrode group; *p < 0.05, **p < 0.01, ***p < 0.001.

RT-PCR was used to evaluate the muscle proteolytic signals of atrogin-1 and Murf1 after chronic stimulation. Atrogin-1 and Murf1 in TA were significantly higher in the DEN and DEN + ES groups compared to those in the CONT group. Atrogin-1 showed a trend toward suppression in the DEN + ES group compared to those in the DEN group, and Murf1 was significantly suppressed in the DEN + ES group compared to those in the DEN group. Atrogin-1 and Murf1 were significantly higher in the DEN and DEN + ES groups of GAS than those in the CONT group and were significantly suppressed in the DEN + ES group compared to those in the DEN group (Fig. 7).

These results indicate that muscle proteolysis, enhanced by the denervation of multiple muscle groups, may be suppressed by B-SES tetanus.

## Discussion

In the acute part of this study, we confirmed that B-SES tetanus stimulation in rats increased glucose consumption and p70S6K phosphorylation in the TA and GAS of both legs. We confirmed that seven days of B-SES treatment suppressed denervation-induced muscle atrophy in multiple muscle groups for the chronic treatment. In addition, B-SES tetanus stimulation in rats with muscle atrophy caused by denervation increased the ribosomal mass and reduced muscle protein degradation. Based on these observations, we investigated whether B-SES-induced tetanus activates multiple skeletal muscles and prevents denervation-induced muscle atrophy by enhancing muscle protein synthesis and inhibiting muscle proteolysis.

The application of B-SES in clinical settings to prevent the loss of skeletal muscle mass has been described in the Introduction. B-SES has been reported to inhibit muscle atrophy and improve motor function in patients by simultaneously activating multiple muscles^7–9^. This study showed that tetanus decreased muscle glycogen content (TA and GAS) and phosphorylation of p70S6K. Glycogen consumption was used as a measure of energy expenditure^24^. As an indicator of mTORC1 activity, p70S6K phosphorylation is believed to improve the efficiency of muscle protein translation, leading to protein synthesis and muscle hypertrophy^4,5^. This was an indicator of training-induced hypertrophy^3,25,26^. In this study, p70S6K phosphorylation significantly increased in both the TA and GAS after a single B-SES stimulation. Tetanus induced by electrical stimulation mimics resistance training, which induces the phosphorylation of p70S6K^3–5^. The acute test showed that B-SES-induced tetanus stimulation resulted in approximately 80% glycogen consumption and a fivefold increase in the phosphorylation of p70S6K in the TA and GAS, the anteroposterior muscles of the lower limb. These results suggested that this study's B-SES tetanus stimulation conditions sufficiently activated multiple muscles and enhanced mTORC1 activity.

In clinical practice, B-SES has been used to prevent muscle atrophy during disuse^7–9^. Although both the twitch and tetanus contractions are used in clinical practice, the effects of different contractions on muscle atrophy in multiple muscle groups remain unknown. Our previous studies in rats confirmed that twitch contraction with B-SES prevented muscle atrophy^6^. In this study, we investigated whether B-SES tetanus stimulation has a preventive effect on muscle atrophy. Denervation in rats results in significant reductions in muscle weight and CSA of both the TA and GAS muscles^2,27,28^. Compared with those in the CONT group, we observed significant reductions in muscle weight and CSA in the DEN group. When denervated rats were subjected to B-SES-induced tetanus, the TA and GAS muscle weights and muscle fiber CSA were significantly higher compared to denervated- treated muscles, suggesting that B-SES simultaneously inhibits muscle atrophy in multiple muscle groups.

After chronic stimulation, the muscles were subjected to RNA-seq to identify differences between the CONT, DEN, and DEN + ES groups. RNA was isolated, and variable genes and pathways were evaluated using RNA-seq-based analysis of differences in gene expression. Mitochondrial adaptation and enhanced ribosome biogenesis have been reported using RNA-Seq upon tetanus contraction stimulation in murine C2C12 myotubes^29^. The RNA-seq results of this study suggest that mitochondria-related genes, mTORC, and the ubiquitin–proteasome pathway are affected. RNA-seq results showed that Rpl4 and Rpl27 gene expression decreased. However, as discussed later, ribosome content was significantly increased in the DEN + ES group than that in the DEN group, suggesting that B-SES may increase ribosome content. Overall, the transcriptional response to electrical stimulation may not coincide with protein levels.

Twitch contractions, as previously reported, inhibit the decrease in mitochondrial synthesis and quantity induced by denervation^6^. In this study, chronic tetanic electrical stimulation with B-SES showed no effect on decreased mitochondrial production (PGC-1α) and quantity (COXIV) despite the enhancement of mitochondrial pathways by RNA-seq. Electrically stimulated tetanus and twitch selectively activated resistance exercise (PKB-TSC2-mTOR signaling) and aerobic exercise (AMPK-PGC-1α signaling) in animal models^5^. However, increased mitochondrial production and quantity have been reported in tetanus^30^, and these metabolic conditions may potentially affect mitochondria. In this study, continuous electrical stimulation was applied for 5 min. In contrast, five sets of 30 s electrical stimulations were applied at 180 s intervals in a previous study^30^. In this study, PKB-TSC2-mTOR signaling was selectively activated under tetanus conditions, while mitochondrial gene expression was increased and may have had little effect on the final production of mitochondria.

Skeletal muscle mass is thought to be regulated by the balance between muscle protein synthesis and degradation^21–23^. Muscle atrophy generally induces proteolytic signals^31^; however, protein synthesis is not necessarily downregulated^32^. Furthermore, repetitive exercise inhibits the phosphorylation of p70S6K, a downstream target of mTOR^33^. Thus, the signaling pathways are not directly related to protein synthesis. Because ribosome content is directly related to protein synthesis, we investigated ribosome biogenesis and quantity to assess muscle protein synthesis. Ribosome quantity has been suggested to be related to muscle protein synthesis and mass^13,14^. Tetanus EMS activates ribosome biogenesis and increases ribosome quantity^15^. The RNA-seq results showed that ribosome biogenesis was downregulated. Western blot analysis of UBF, TIF-1A, and c-myc, which are involved in ribosome biosynthesis, showed a significant increase in UBF in TA in the DEN and DEN + ES groups and no significant differences among the three groups in the other muscle groups. Further, ribosomal abundance was examined using 18S, 28S, and the precursor 45S (45Spre-rRNA). The results showed a significant increase in 18S and 28S rRNA in the DEN + ES group compared to those in the DEN group in both TA and GAS. 45S rRNA was not significantly different between the DEN and DEN + ES groups in GAS and showed a significant trend in TA. Electrical stimulation increased ribosome contents without any change or even a decrease in ribosome biogenesis.

We evaluated ribosome biogenesis and content and observed an increase in UBF; however, few significant changes were observed in TIF-1A and c-myc, which signal ribosome biogenesis due to denervation and electrical stimulation. Similar results have been reported for the ribosome synthesis signal induced by denervation^34^. However, in this study, tetanic contractions could not rescue these changes induced by denervation. However, an increase in ribosome content was observed upon tetanic contraction, suggesting an increase in muscle protein synthesis. Muscle contractions induced by electrical stimulation increase ribosome content. However, signals such as UBF, TIF-1a, and c-myc may vary with the number of electrical stimulations and the analysis timing^10^. This suggests that the results of this study may not show any change in ribosome synthesis signals, depending on the conditions of electrical stimulation and the timing of the analysis.

Differences were observed between mitochondria-related and ribosome-related gene expression obtained by RNAseq in this study and actual protein expression in Western blots. As noted above, this variability may be due to electrical stimulation conditions, muscle sampling for analysis, and timing of analysis^35^. In addition, differences in translation efficiency, which are not visible in mRNA abundance measurements, contribute significantly to the dynamic range of gene expression, and protein-level measurements at the mRNA level are considered incomplete^36^, which may have led to the results of this study.

Atrogin-1 and MuRF1 regulate muscle size by promoting muscle protein loss through protein degradation^37–39^. These signals were enhanced by denervation and inhibited by the electrical stimulation of tetanus^39^. Therefore, we evaluated the mRNA levels of atrogin-1 and Murf1, which are muscle proteolytic signals. We observed that denervation significantly increased the mRNA expression of Atrogin-1, and Murf1. The increase was significantly suppressed or tended to be suppressed by B-SES. These results suggest that B-SES may inhibit muscle proteolysis in TA and GAS in B-SES-induced tetanus. In conclusion, this study suggests that 2-electrode B-SES-induced tetanus suppresses muscle atrophy in multiple muscle groups and that promoting muscle protein synthesis and suppressing muscle protein degradation may be one factor.

As previously reported, a twitch induced by the same 2-electrode B-SES system activates multiple muscle groups with twitch contractions and suppresses denervation-induced muscle atrophy in multiple muscle groups by maintaining mitochondrial mass, enzyme activity, and suppressing muscle protein degradation^6^. These results are consistent with previous studies showing that electrical stimulation of tetanus and twitch selectively activates muscle protein synthesis (PKB-TSC2-mTOR signaling, mimicking resistance exercise) and mitochondrial synthesis (AMPK-PGC-1α signaling, mimicking aerobic exercise) in animal models, respectively^5^. This finding supports the hypothesis that stimulation with B-SES has similar effects on multiple muscle groups. Although muscle atrophy was suppressed in both contraction modes, it would be desirable to use TWITCH when the goal is to increase energy metabolism, as in aerobic training, or TETANUS when the goal is to improve muscle mass and maintain muscle strength, as in resistance training, and to adjust the contraction mode to the exercise when combining exercise and electrical stimulation^40^. Overall, the results suggest that selecting the appropriate B-SES contraction mode is promising for the efficient use of EMS in clinical situations.

## Methods

### Animals

Ten-week-old male Sprague–Dawley rats were purchased from CLEA (Tokyo, Japan). The rats were housed in cages at 23℃ with a 12 h/12 h light/dark cycle (dark time 18:00–06:00). All rats received a standard solid diet (CE-7; CLEA Japan) and water ad libitum. The mean body weight of all animals was 345 ± 24 g (mean ± SD). All rats were acclimatized by rearing in the environment described above for 1 week before the experiment. Both acute and chronic belt electrode experiments were conducted in this study. All animals were randomly assigned as follows: (1) acute stimulation experiment, N = 12; (2) chronic stimulation experiment, N = 30.

### Electrical stimulation with belt electrodes

The right and left ankle joints were shaved under isoflurane anesthesia (anesthetic aspiration rate: 250–300 mL/min, concentration: 2.0–2.2%). The rats were placed on their backs on a table, and belt-type electrodes (Homer Ion Co., Tokyo) were attached to both ankle joints.

The electrical stimulation method was modified from a previous study^33^. The muscles of the lower extremities were stimulated simultaneously on both sides using electrical stimulation at 60 Hz, eliciting continuous tetanus for 5 min. Stimulation intensity was preliminarily tested with a belt electrode and set to 3.0 mA, which is the minimum current run value at which maximum torque was obtained with 60 Hz stimulation. During contraction, the lower limb was not immobilized, and the exercise was performed in a natural extension position.

### Acute response to belt electrode electrical stimulation

**Figure 8:**
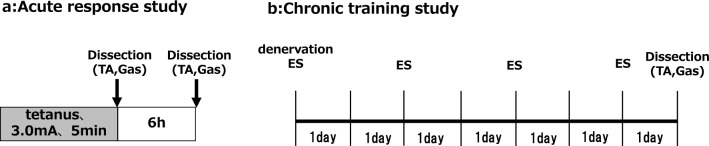
(**a**) Acute and (**b**) chronic stimulation study design. Acute medial GAS and TA muscles were dissected immediately (glycogen content analysis) or six hours (p70S6K analysis) after stimulation to assess muscle glycogen content and phosphorylated p70S6K. Chronically stimulated TA and GAS muscles were dissected 24 h after the last stimulus of the chronic response. Both muscles were used for muscle weight, CSA, western blotting, RT-PCR, and muscle glycogen content analysis.

An acute response analysis was performed for the CONT and electrically stimulated (ES) groups. Under isoflurane anesthesia, a single stimulation (1 set of 5 min, Fig. 8a) with a belt electrode was performed. In addition, glycogen levels were assessed by excising the TA and GAS muscles immediately after exercise, and the TA and GAS muscles were removed six hours after exercise to measure phosphorylated p70S6K (n = 6).

### Chronic response to belt electrode electrical stimulation

Chronic responses to belt electrode stimulation were divided into three groups: control (CONT), denervation (DEN), and denervation + electrical stimulation (DEN + ES), and a chronic response analysis was performed. For electrical stimulation, chronic responses (5 min × 1 set, once every two days, for seven days) were performed under isoflurane anesthesia, and TA and GAS were harvested 24 h after the last stimulation (Fig. 8b).

Muscle weight (n = 8–11), muscle atrophy inhibition by muscle fiber cross-sectional area (CSA) (n = 6), RNA-seq, mitochondrial synthesis, quantity signaling, ribosome biogenesis, and quantity (n = 6) indices were evaluated.

### Denervation

Since sciatic nerve denervation induces muscle atrophy, surgical denervation was performed^41^. Under isoflurane anesthesia, the sciatic nerve was exposed through a small incision on the posterior aspect of the right and left hind limbs. The sciatic nerve was excised using small surgical scissors and the skin, was sealed using surgical glue. After denervation, a lack of mobility was observed in both legs, where the nerves were severed.

### Skeletal muscle analysis

TA and GAS were used in the experiments. The resulting muscle was cut into two pieces for biochemical analysis and CSA measurement of the muscle fibers.

For CSA measurements, TA and GAS muscles were embedded in Optimal Cutting Temperature (O.C.T) compound (Sakura Finetech Japan, Tokyo, Japan) and flash frozen in cooled isopentane (166-00615, Fujifilm Wako Pure Chemicals Corporation, Osaka, Japan). Additional TA and GAS muscles were flash-frozen in liquid nitrogen for western blotting, PCR, and muscle glycogen content analysis. All muscle samples were stored at -80 °C until analysis.

### Muscle glycogen content

Frozen muscle (10–20 mg) was powdered and diluted in a homogenization buffer containing 300 µL 30% KOH saturated with 100 µL 1 M Na2SO4. Samples were boiled at 95 °C for 30 min with mixing every 10 min. After incubation, 480 µL of ethanol was added, and the mixture was centrifuged at 1000× g for 5 min at 4℃. The supernatant was discarded, and the pellet was dried for 1 h. Finally, glycogen content was determined by measuring the absorbance at 505 nm using the Lab Assay TM Glucose Kit (298-65701, Fujifilm Wako Pure Chemicals Corp, Osaka, Japan). The obtained data were corrected for the weight of each muscle in powder form.

### Cross-sectional area of muscle fiber

Experimental methods and CSA analysis were based on previous studies^42–44^. The GAS and TA muscles were cut into 10-μm-thick frozen sections using a cryostat (CM-502, Sakura Finetech). Samples were blocked with 5% goat serum (PCN5000, Thermo Fisher Scientific Japan, Tokyo, Japan) for 1 h, incubated with a primary anti-laminin antibody (L8271, 1:1000, Sigma Aldrich Japan, Tokyo, Japan) for 2 h. After incubation, the cells were washed with 0.1 M phosphate buffer (5 min × 3 times) and incubated with Alexa fluor 488 conjugated secondary antibody (1:2000, A-11008, Thermo Fisher Scientific, MA, USA) for 2 h at room temperature. Images were captured using a confocal laser microscope (FV-3000; Olympus, Tokyo, Japan) and quantified using MyoVision (University of Kentucky). Twelve images were acquired from four sections per muscle for analysis using approximately 4500 to 8000 fibers per group.

### Protein extraction and western blotting

Western blotting was performed as previously described^45–47^. Muscle samples were homogenized in radioimmunoprecipitation assay buffer (188-02453, Fujifilm Corporation) containing a protease and phosphatase inhibitor cocktail (169-26063/167-24381, Fujifilm Wako Pure Chemicals Co.). Protein concentrations of the samples were determined using the BCA method (297-73101, Fujifilm Wako Pure Chemicals Co.). Equal amounts (40 μg) of protein were separated by SDS-PAGE (NW04125BOX, Thermo Fisher Scientific) and transferred to a polyvinylidene fluoride membrane (IB24001, Thermo Fisher Scientific). Protein transfer was confirmed using Ponceau S staining (33427.01; SERVA Electrophoresis GmbH, Heidelberg, Germany). The membrane was cut at the site of the desired molecular weight and subsequent reactions were performed (supplement information). Membranes were blocked with a blocking reagent (NYPBR01, Toyobo, Osaka, Japan) for 1 h, and primary antibodies (Table 2) were diluted with a dilution reagent (NKB-101, Toyobo). After incubation, the cells were washed with Tris-buffered saline containing 0.01% Tween-20 (TBST; T9142, Takara Bio Inc.). The membrane was then incubated with a secondary antibody (7074/7076 Cell Signaling Technology) diluted with reagent (NKB-101, Toyobo) for 1 h at room temperature and washed again with TBST. Protein bands were visualized using a fluorescent reagent (SuperSignal West Pico Chemiluminescent Substrate; Thermo Fisher Scientific). iBright 1500 (FL1500, Thermo Fisher Scientific) and iBright Analysis Software (Windows, Thermo Fisher Scientific) were used to scan and quantify the blots. Ponceau S signal intensity was used as a loading control.

**Table 2:** Antibodies used in Western Blot analysis.

Protein	Suppier	Product No
Phospho-p70 S6 Kinase (Thr389)	Cell Signaling Technology	9205
p70 S6 Kinase	Cell Signaling Technology	9202
c-myc	Cell Signaling Technology	5605
TIF-IA	Santa Cruz Biotechnology	sc-390464
UBF	Santa Cruz Biotechnology	sc-13125

### Library preparation, RNA-seq, and bioinformatics analysis

RNA-seq was performed as previously described^29^. Sequencing libraries were prepared using the QuantSeq 30 mRNA-seq LibraryPrep Kit FWD for Illumina (Lexogen, Inc.). After removal of the RNA template, a second strand was synthesized using random primers. The double-stranded cDNA library was purified using magnetic beads after washing, and the library was amplified by PCR using primers containing adapters and i7 index sequences. Libraries were purified with magnetic beads, quantified using an Affluorometer (Qubit 4, Thermo Fisher Scientific), and pooled to equimolar concentrations. Sequencing was performed on a Mini Seq system (Illumina, San Diego, CA, USA) with 75 cycles using the Mini Seq High Output Reagent Kit. After sequencing, sequence quality was examined using FastQC.

Gene expression difference analysis and pathway analysis were then performed by iDEP. Gene expression difference analysis was performed using DEseq2 [false discovery rate (FDR) cutoff: 0.1, minimum fold change: 2]. Pathway analysis was performed on the GO Biological Process gene set using Generally Applicable Gene Set Enrichment for pathway analysis (GAGE; FDR cutoff:0.10).

### Ribosomal RNA amounts

RNA was isolated from tissues as previously reported^15,48^. Following this method, muscle was homogenized with TRIzol reagent (Thermo Fisher Scientific). Chloroform was added, mixed, and allowed to stand for 15 min, and the muscle samples were centrifuged at 4 °C, 12,000× g for 15 min. The supernatant was collected, ethanol was added and mixed, and total RNA was extracted using RNeasy kit (74106; QIAGEN, Hilden, Germany).

RNA solutions were mixed 5:1 with GRR-1000GR Red Loading Buffer (Biocraft, Tokyo, Japan), electrophoresed on 1% agarose (NE-AG; Fast Gene, Tokyo, Japan)-TAE, and 18S and 28S rRNA bands were scanned using ChemiDoc XRS (170–9071, Bio-Rad) and Quantity One software (170–9600, version 4. 5.2, Windows; Bio-Rad) were used to scan. The band intensities were corrected for the muscle wet weight.

### RT-PCR

RT-PCR was performed as previously described^4^. Muscle samples were homogenized using TRIzol reagent (356,203, Thermo Fisher Scientific). Chloroform (163–20,145, Fujifilm Wako Pure Chemicals Corporation) was added to the homogenized samples, mixed, and allowed to stand for 15 min. Muscle samples were centrifuged at 4 °C and 12,000 × g for 15 min. After collecting the supernatant, ethanol was added and mixed. Total RNA was extracted using an RNA extraction kit (74,106; QIAGEN, Hilden, Germany). Total RNA concentration was measured using NanoDrop One (Thermo Fisher Scientific), and 1500 ng of total RNA was extracted using a High-Capacity cDNA RT kit (Applied Biosystems, Foster City, CA, USA). Reverse transcription was performed using cDNA. Real-time PCR was performed using the SYBR gene expression assay (Applied Biosystems) in an optical reaction module equipped with a thermal cycler (CFX96, Bio-Rad, California, USA) using primers (Table 3).

**Table 3:** Primer sequences used in RT qPCR analysis.

Target	Forwad	Reverse
45S pre-rRNA	TGGGGCAGCTTTATGACAAC	TAGCACCAAACGGGAAAACC
Atrogin-1	AAGGAGCGCCATGGATACTG	AGCTCCAACAGCCTTACTACG
Murf1	GACATCTTCCACGCTGCCAA	TGCCGGTCCATGATCACTTC

### Statistical analysis

Data are presented as the mean ± Standard error. Statistical significance was assessed using a parametric unpaired t-test for the acute stimulation study and compared between the two groups. In chronic stimulation, changes in muscle wet weight, CSA measurements, mitochondria-related signals, muscle proteolytic signals, ribosomal synthesis signals, and rRNA levels were evaluated by one-way ANOVA. Using Uncorrected Fisher's LSD correction for multiple comparisons. Statistical significance was set at p < 0.05, and statistical evaluation was performed using Graph statistical analysis software. GraphPad Prism (version 8.3.0; GraphPad Software, San Diego, CA, USA) was used for the statistical analysis.

### Ethical approval

All experiments were approved by the Animal Experimentation Committee of the Japan Sport Sciences University. All experiments complied with the policies and regulations of the “Basic Guidelines for the Appropriate Conduct of Animal Experiments and Related Activities in Academic Research Institutions” issued by the Ministry of Education, Culture, Sports, Science, and Technology of Japan. The study was conducted in accordance with the ARRIVE guidelines.

### Supplementary Information

Supplementary Information.

## Data Availability

The datasets generated and/or analysed during the current study are available in the Array Express repository, E-MTAB-13286.
