# Supplementary material for: Belt electrode tetanus muscle stimulation reduces denervation-induced atrophy of rat multiple skeletal muscle groups
Source: Sci Rep. 2024 Mar 11;14:5848. doi: 10.1038/s41598-024-56382-x (PMC10925608; doi:10.1038/s41598-024-56382-x)

Supplemental figure legend

Figure S1 : Western blot membrane images.
The membrane was cut at the position of the target molecular weight when the antibody reaction.

Figure S4 a,b、S5 a,b,c : Western blot membrane images.
The membrane was cut at the position of the desired molecular weight and the antibody reaction was performed. 2 membranes were photographed simultaneously for analysis.
Red frame: used in the figure.

Figure S6：Ribosomal RNA amounts analysis electrophoresis image.
Red frame: used in the figure

Supplementary Figure S1 : Western blot membrane images of total and phosphorylated p70S6k.

The membrane was cut at the position of the target molecular weight when the antibody reaction.

Tibialis anterior

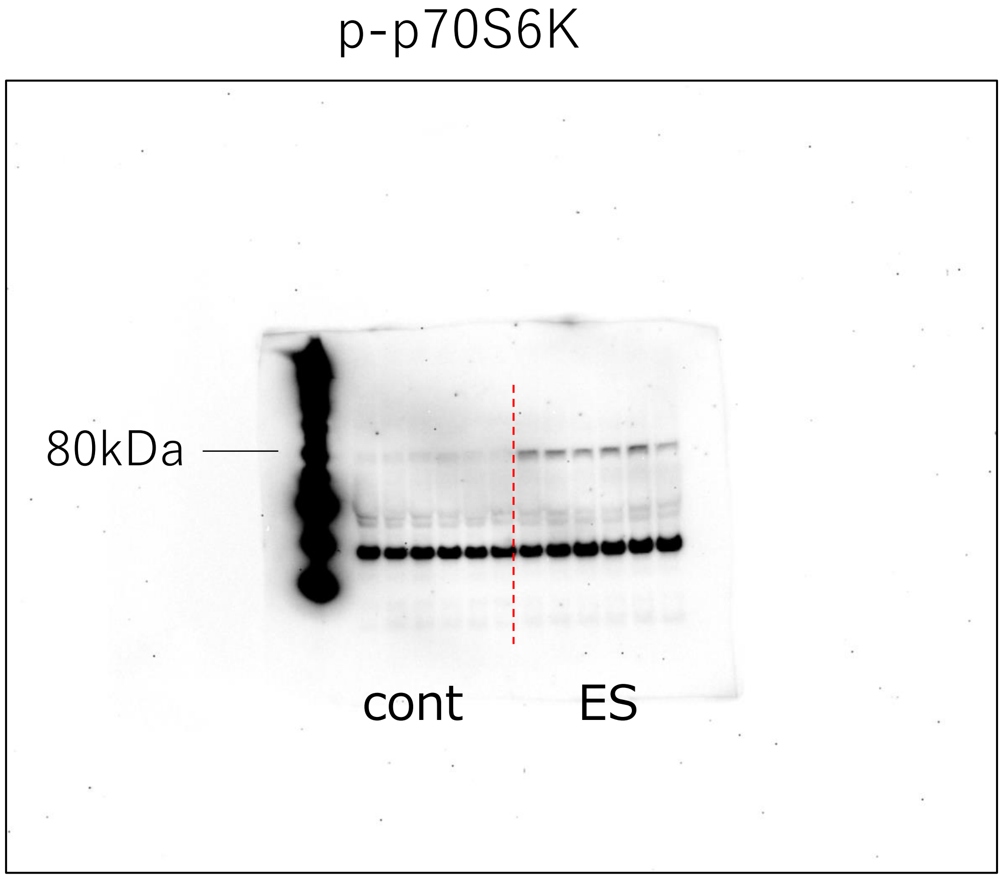

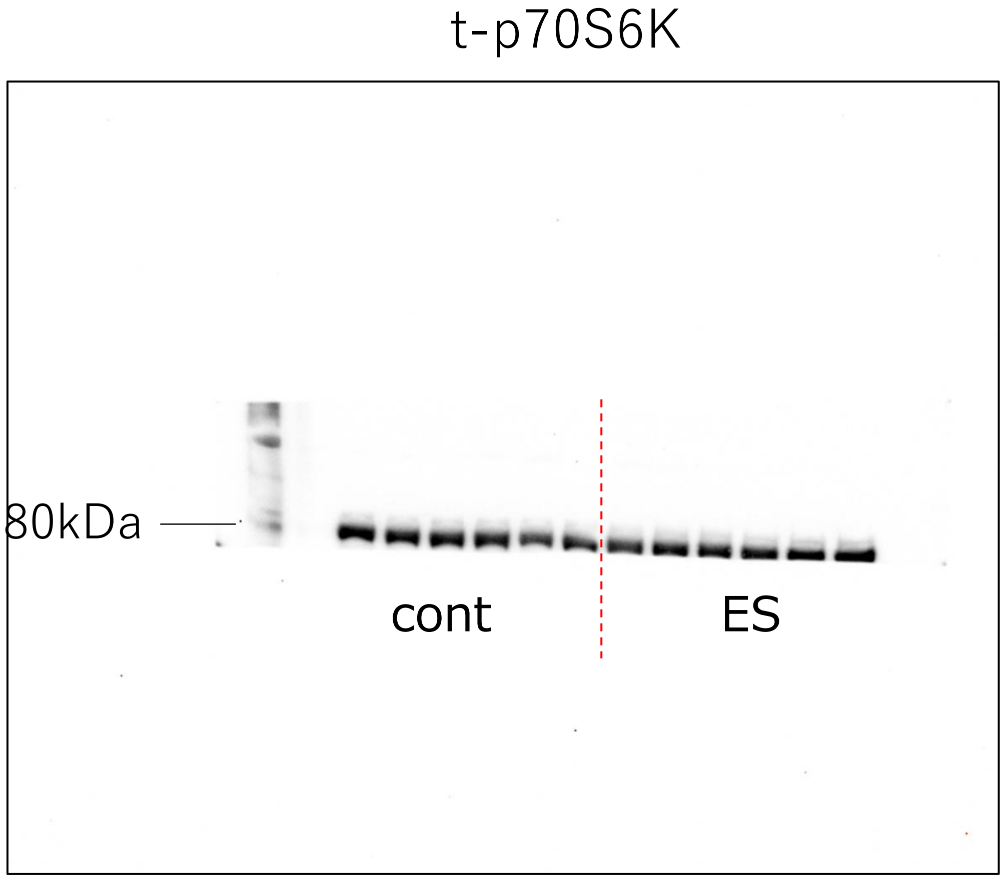

Supplementary Figure S1 : Western blot membrane images of total and phosphorylated p70S6k.

The membrane was cut at the position of the target molecular weight when the antibody reaction.

Gastrocnemius

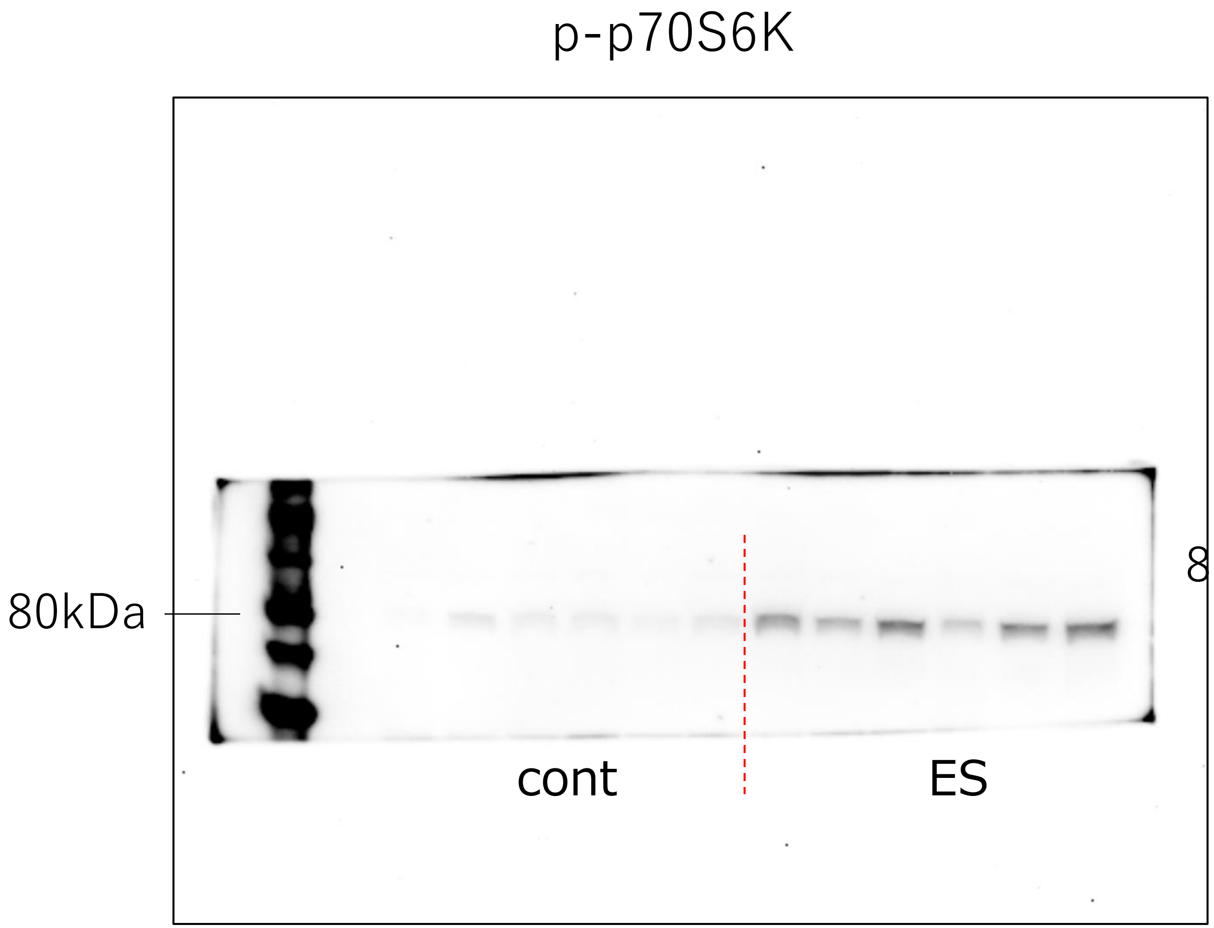

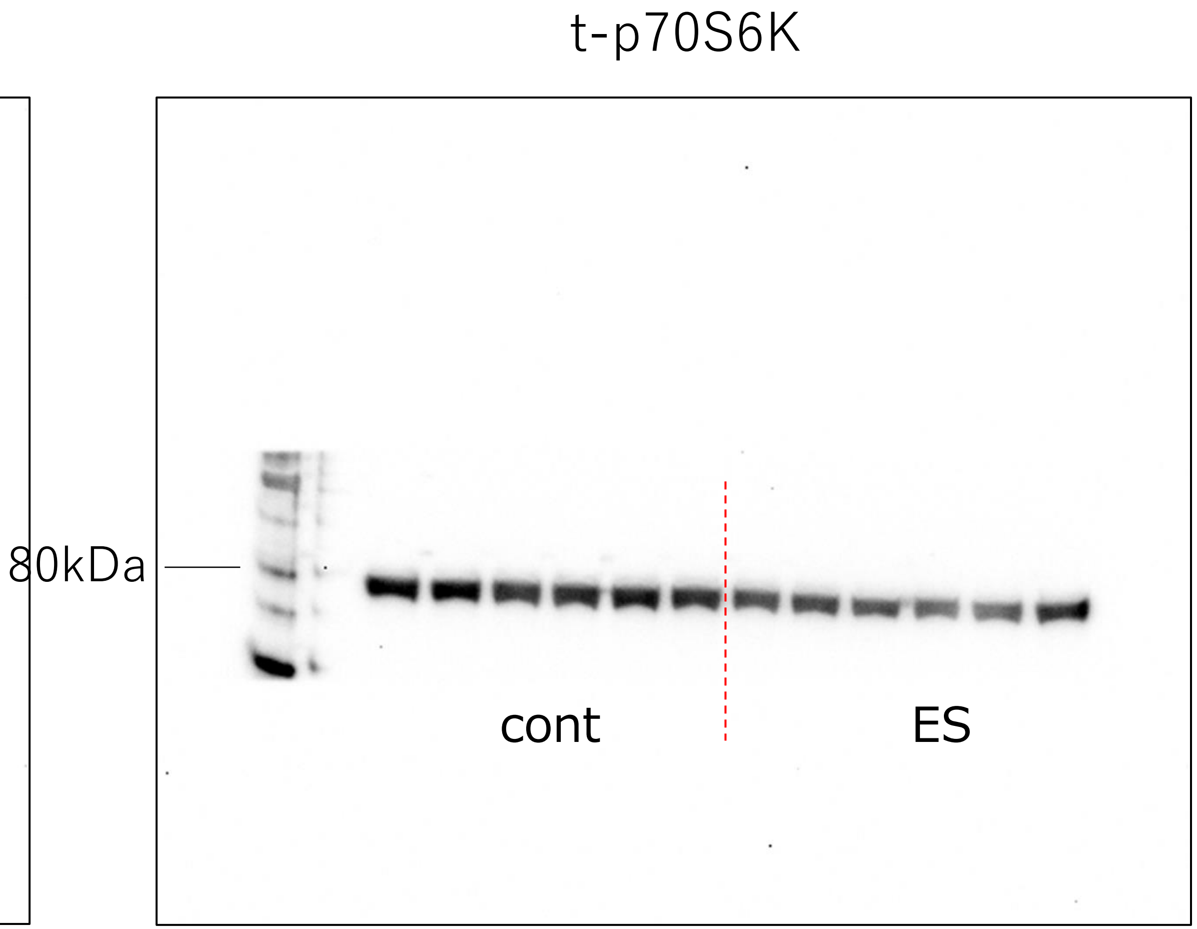

Supplementary Figure S4 a: Western blot membrane images of PGC-1α.

The membrane was cut at the position of the desired molecular weight and the antibody reaction was performed. 2 membranes were photographed simultaneously for analysis.

Red frame: used in the figure.

Tibialis anterior

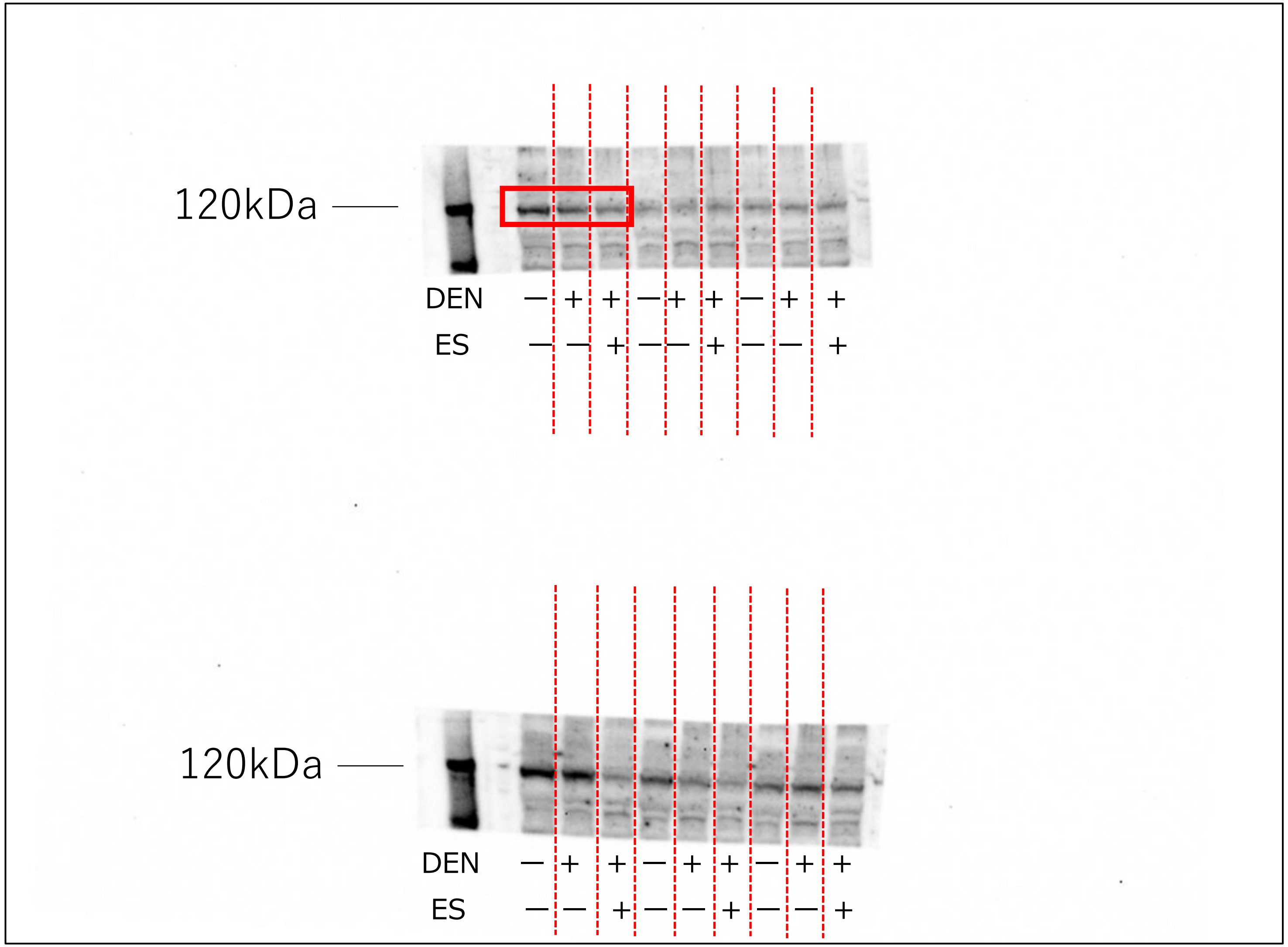

Supplementary Figure S4 a: Western blot membrane images of PGC-1α.

The membrane was cut at the position of the desired molecular weight and the antibody reaction was performed. 2 membranes were photographed simultaneously for analysis.

Red frame: used in the figure.

Gastrocnemius

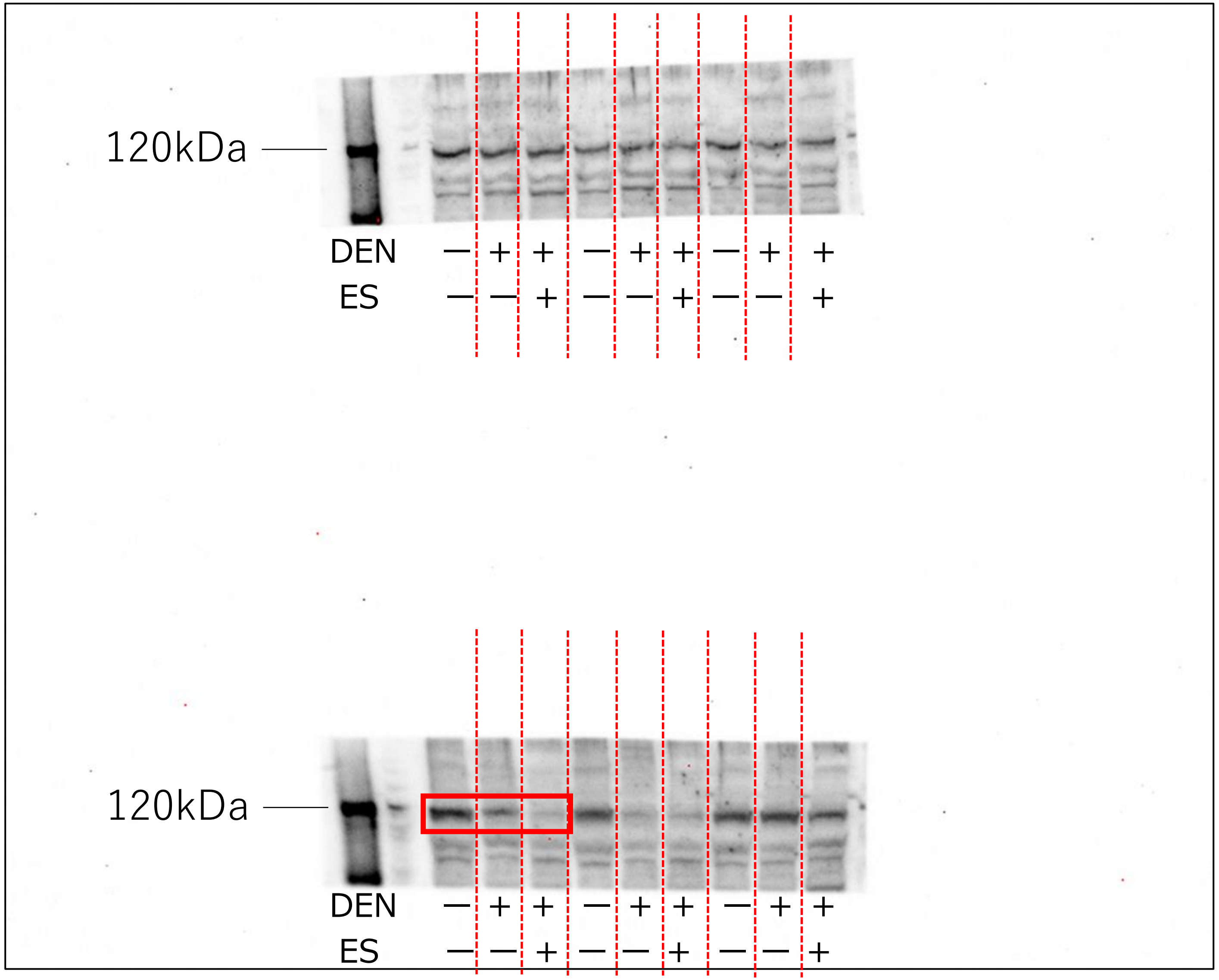

Supplementary Figure S4 b: Western blot membrane images of COXIV.

The membrane was cut at the position of the desired molecular weight and the antibody reaction was performed. 2 membranes were photographed simultaneously for analysis.

Red frame: used in the figure.

Tibialis anterior

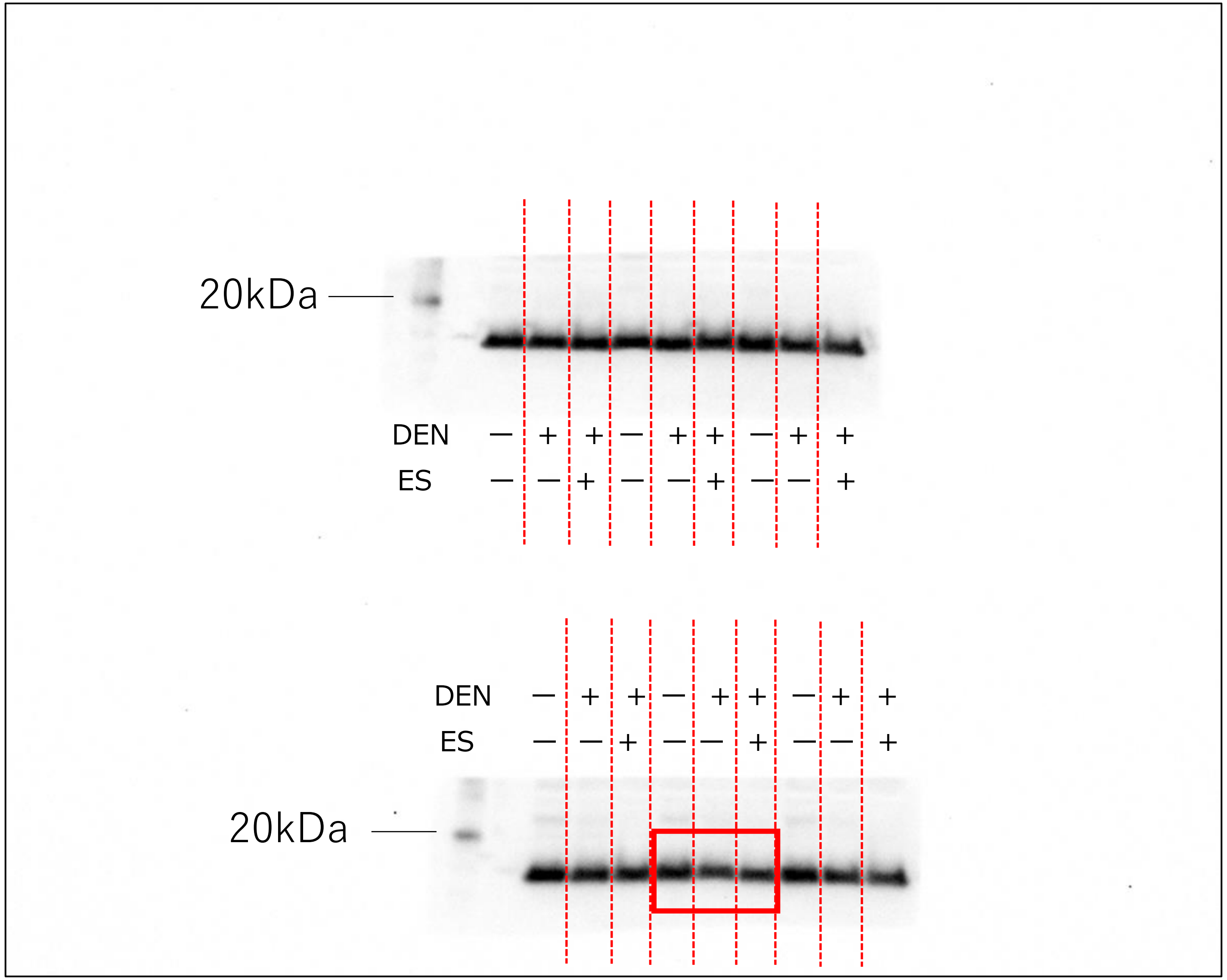

Supplementary Figure S4 b: Western blot membrane images of COXIV multipe exposuretime.

Tibialis anterior

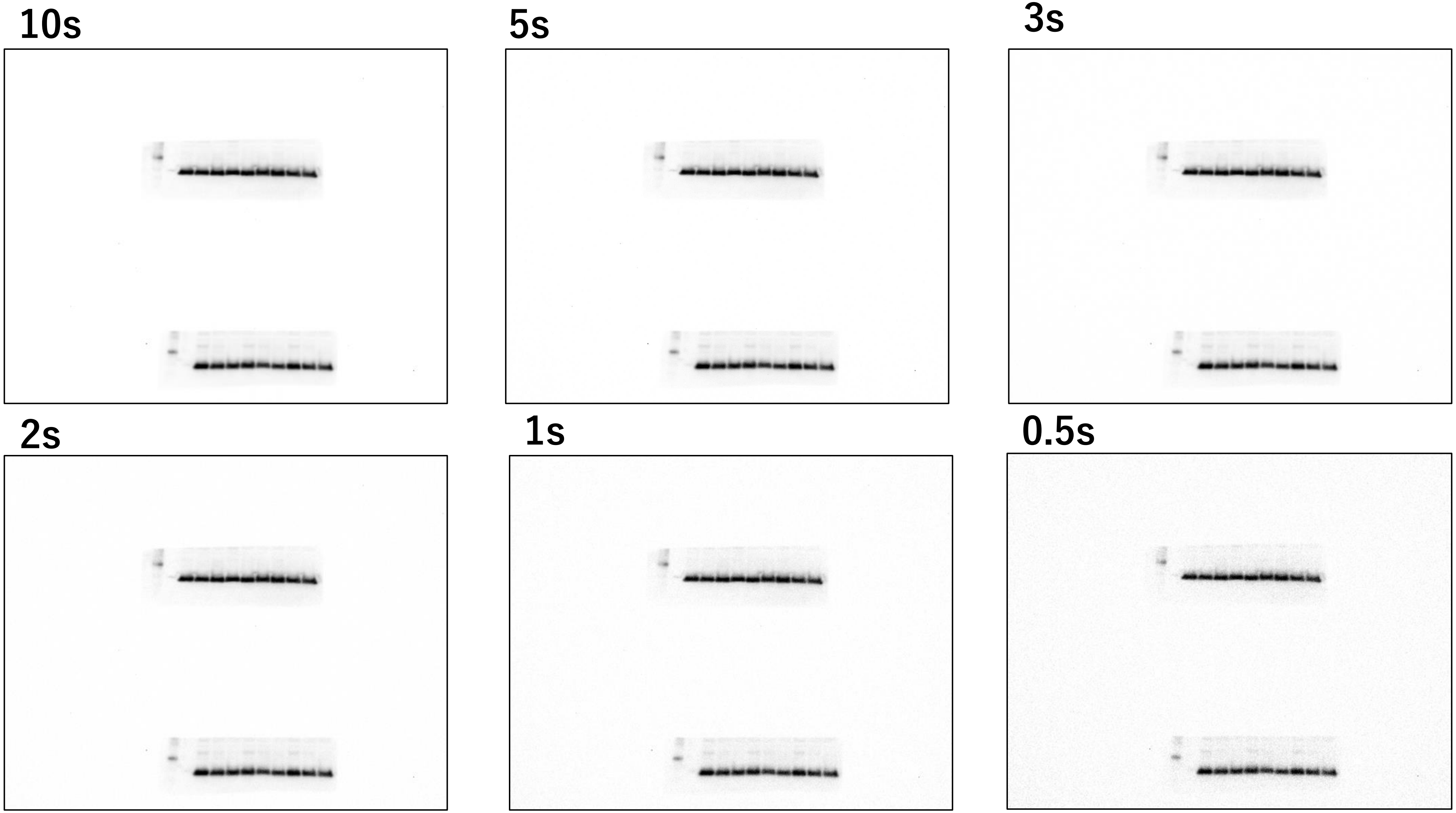

Supplementary Figure S4 b: Western blot membrane images of COXIV.

The membrane was cut at the position of the desired molecular weight and the antibody reaction was performed. 2 membranes were photographed simultaneously for analysis.

Red frame: used in the figure.

Gastrocnemius

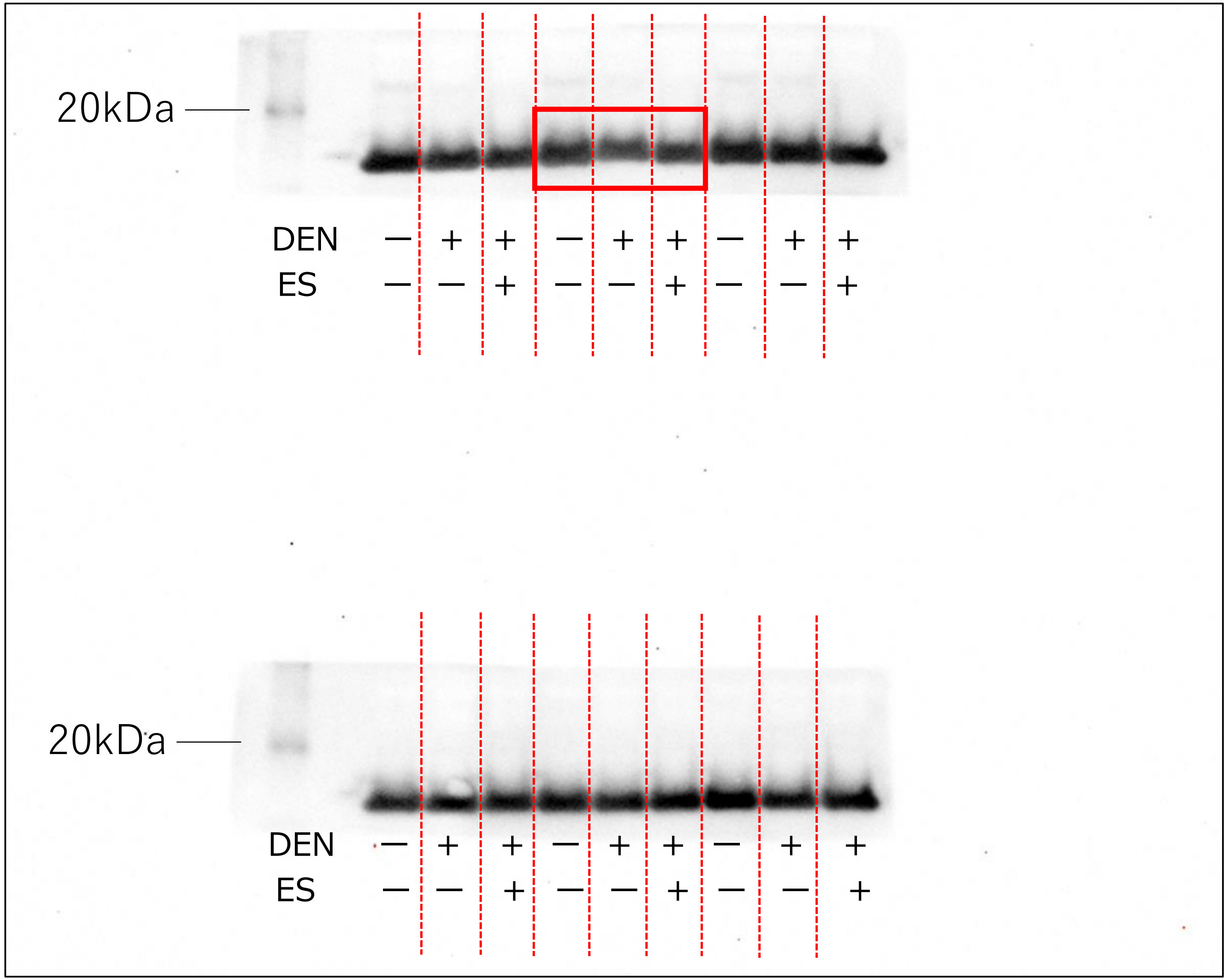

Supplementary Figure S4 b: Western blot membrane images of COXIV multipe exposuretime.

Gastrocnemius

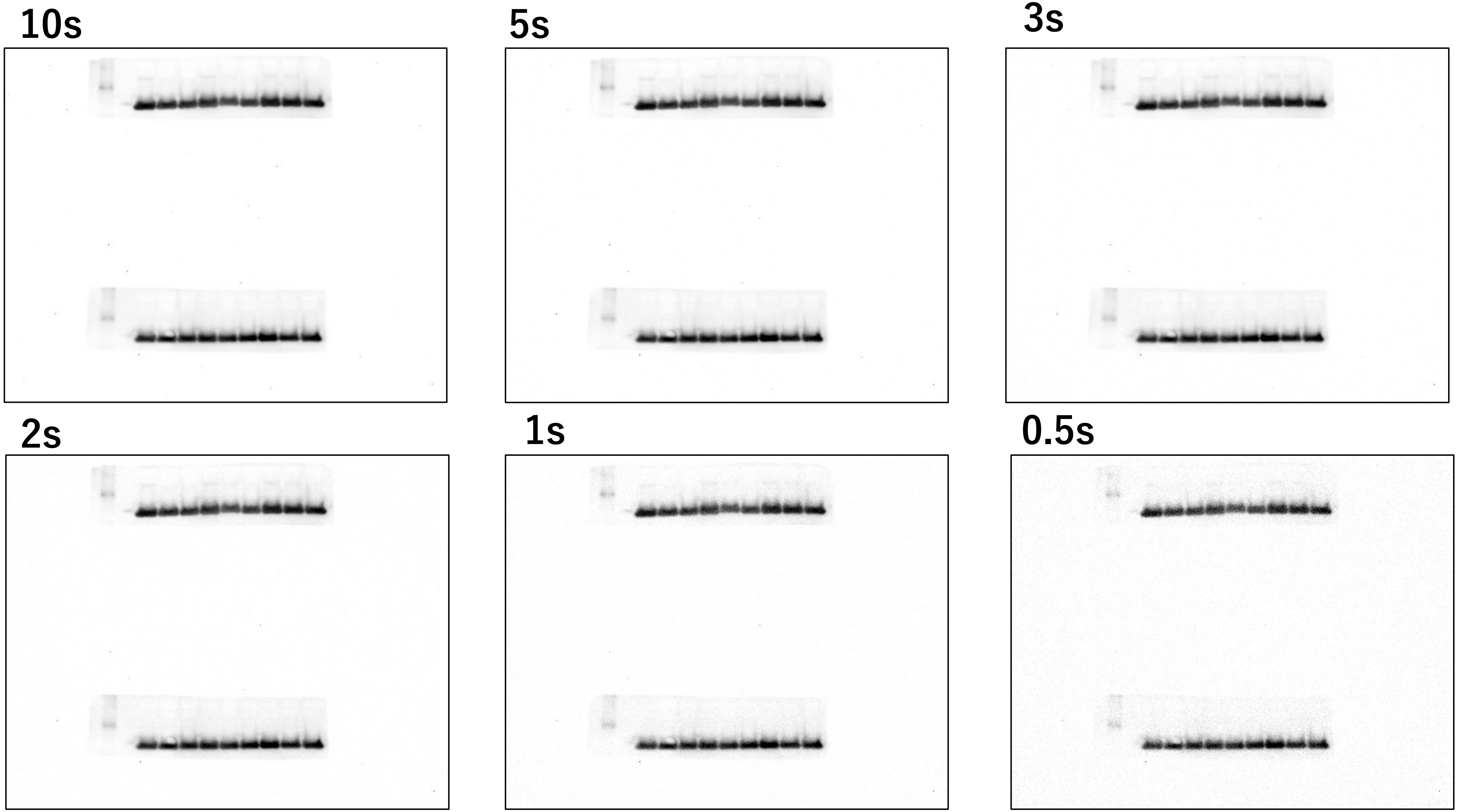

Supplementary Figure S5：Ribosomal RNA amounts analysis electrophoresis image.

Red frame: used in the figure

Tibialis anterior

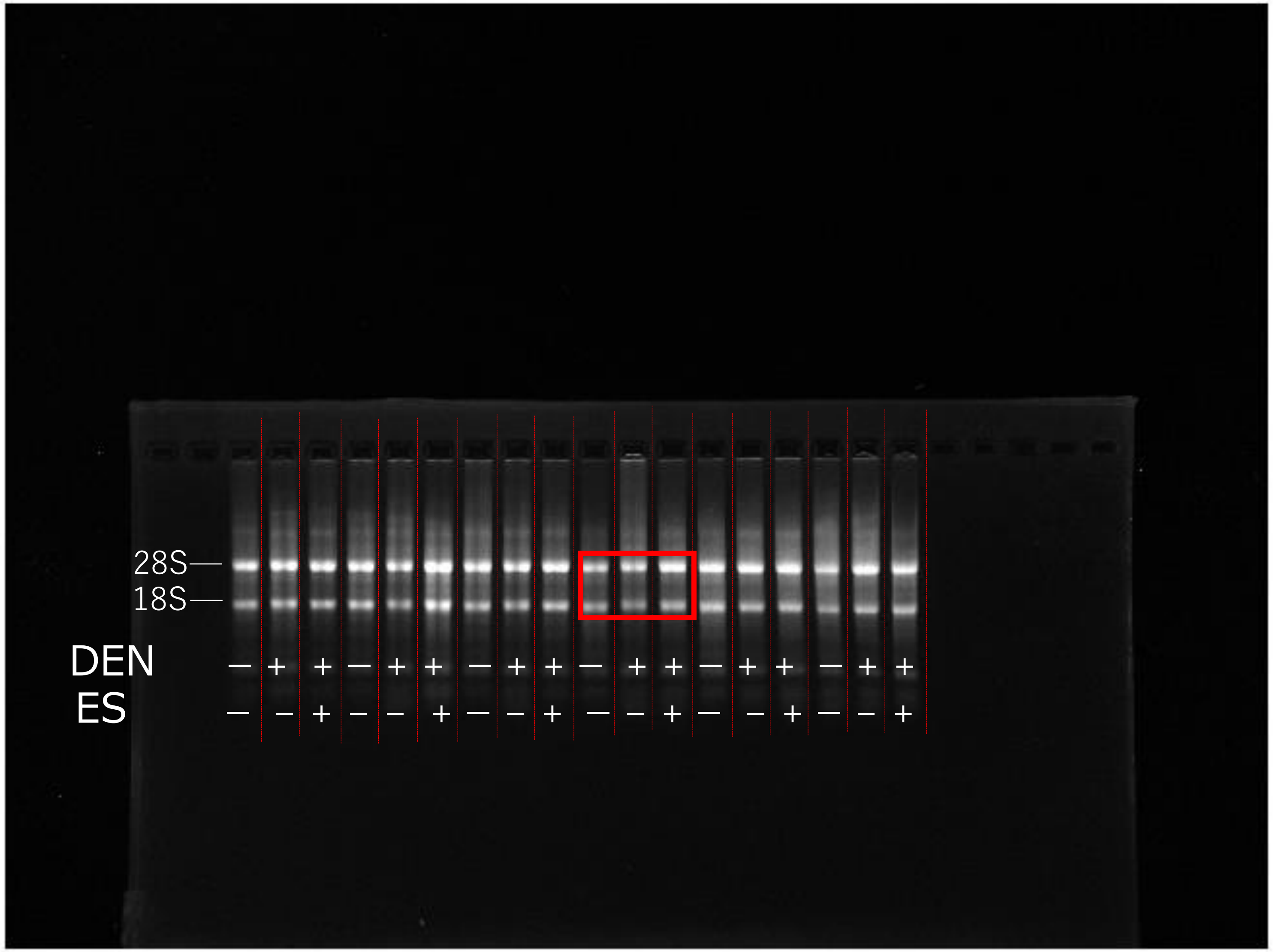

Supplementary Figure S5：Ribosomal RNA amounts analysis electrophoresis image.

Red frame: used in the figure

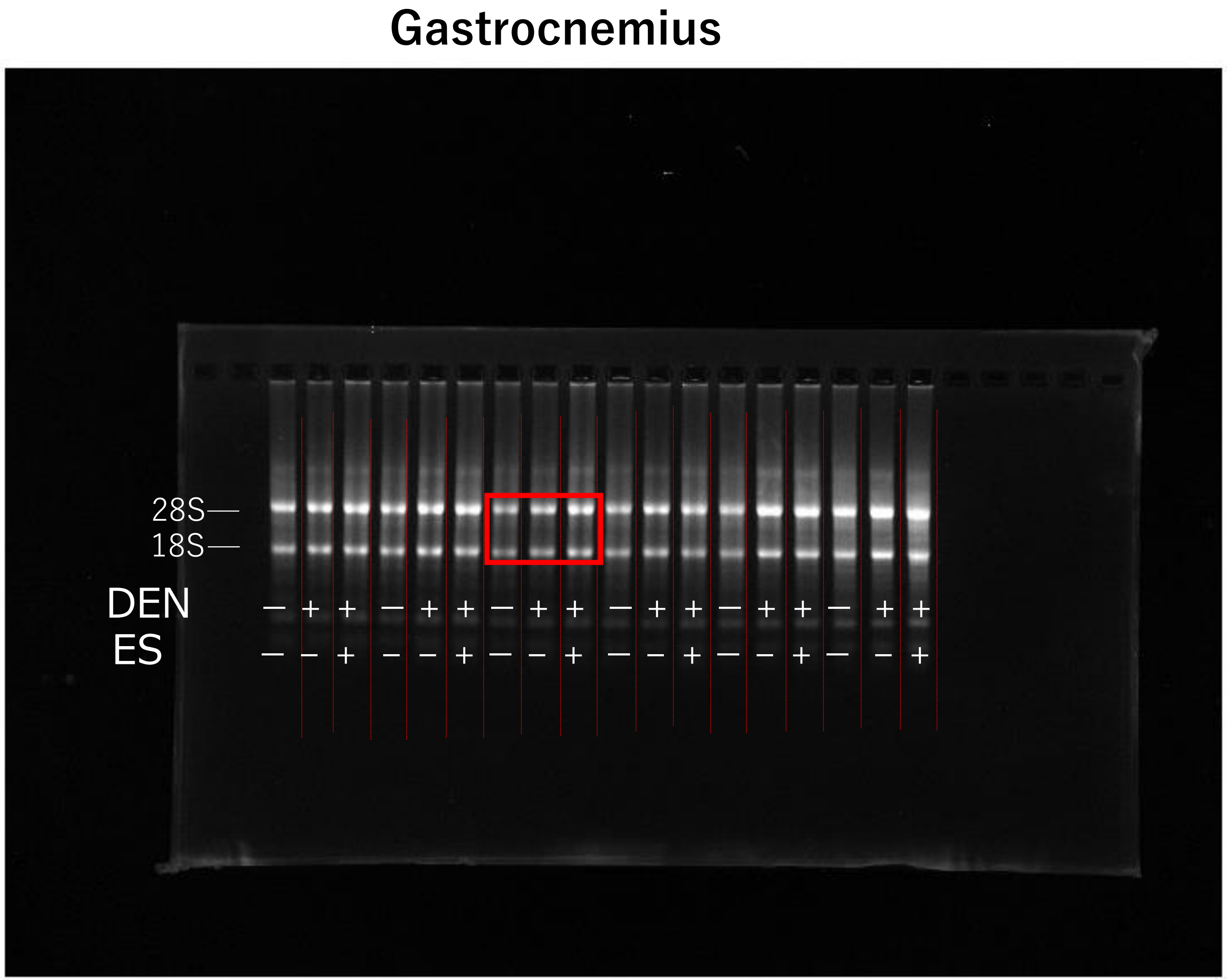

Supplementary Figure S6 a: Western blot membrane images of UBF.

The membrane was cut at the position of the desired molecular weight and the antibody reaction was performed. 2 membranes were photographed simultaneously for analysis.

Red frame: used in the figure.

Tibialis anterior

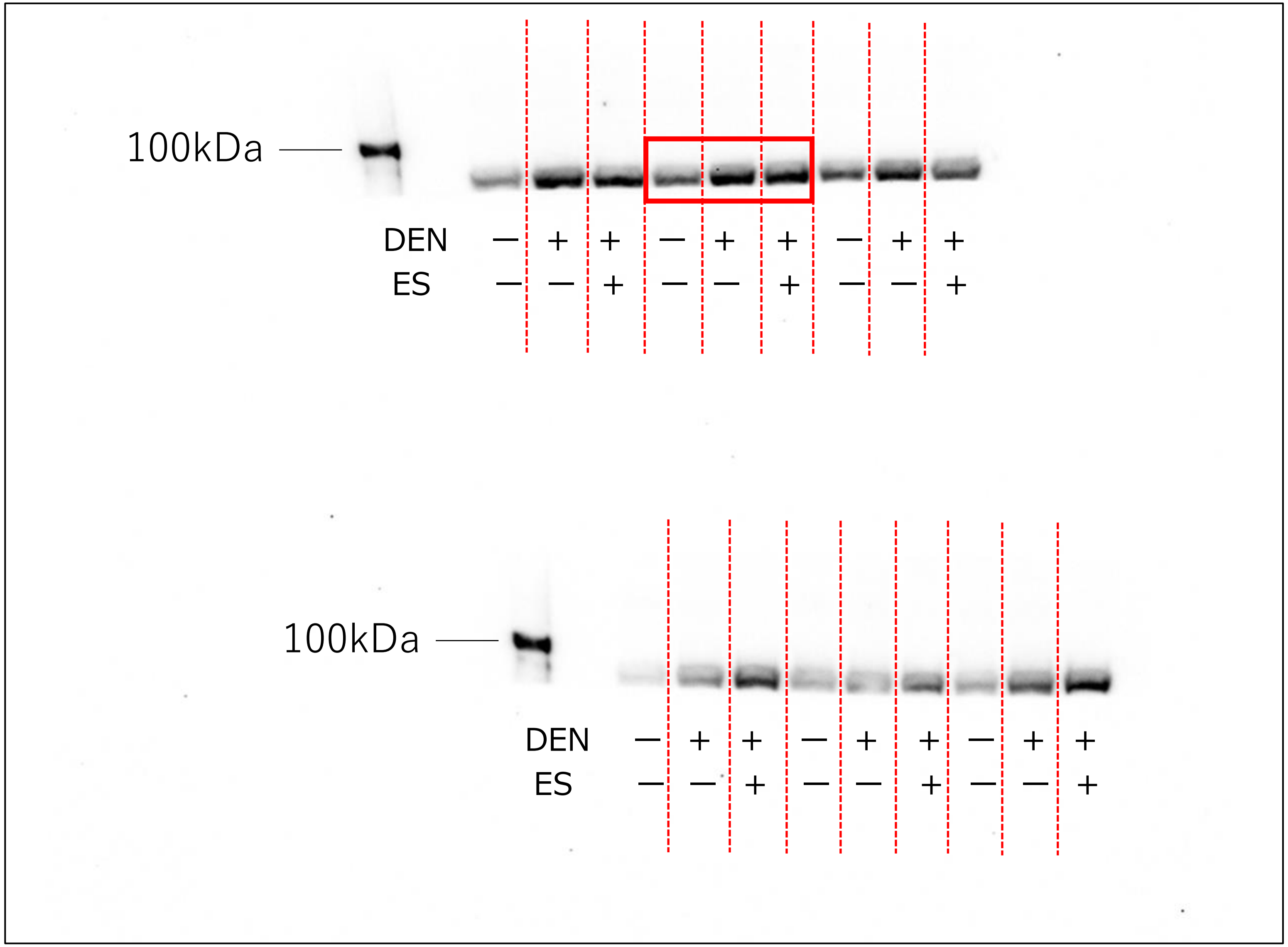

Supplementary Figure S6 a: Western blot membrane images of UBF.

The membrane was cut at the position of the desired molecular weight and the antibody reaction was performed. 2 membranes were photographed simultaneously for analysis.

Red frame: used in the figure.

Gastrocnemius

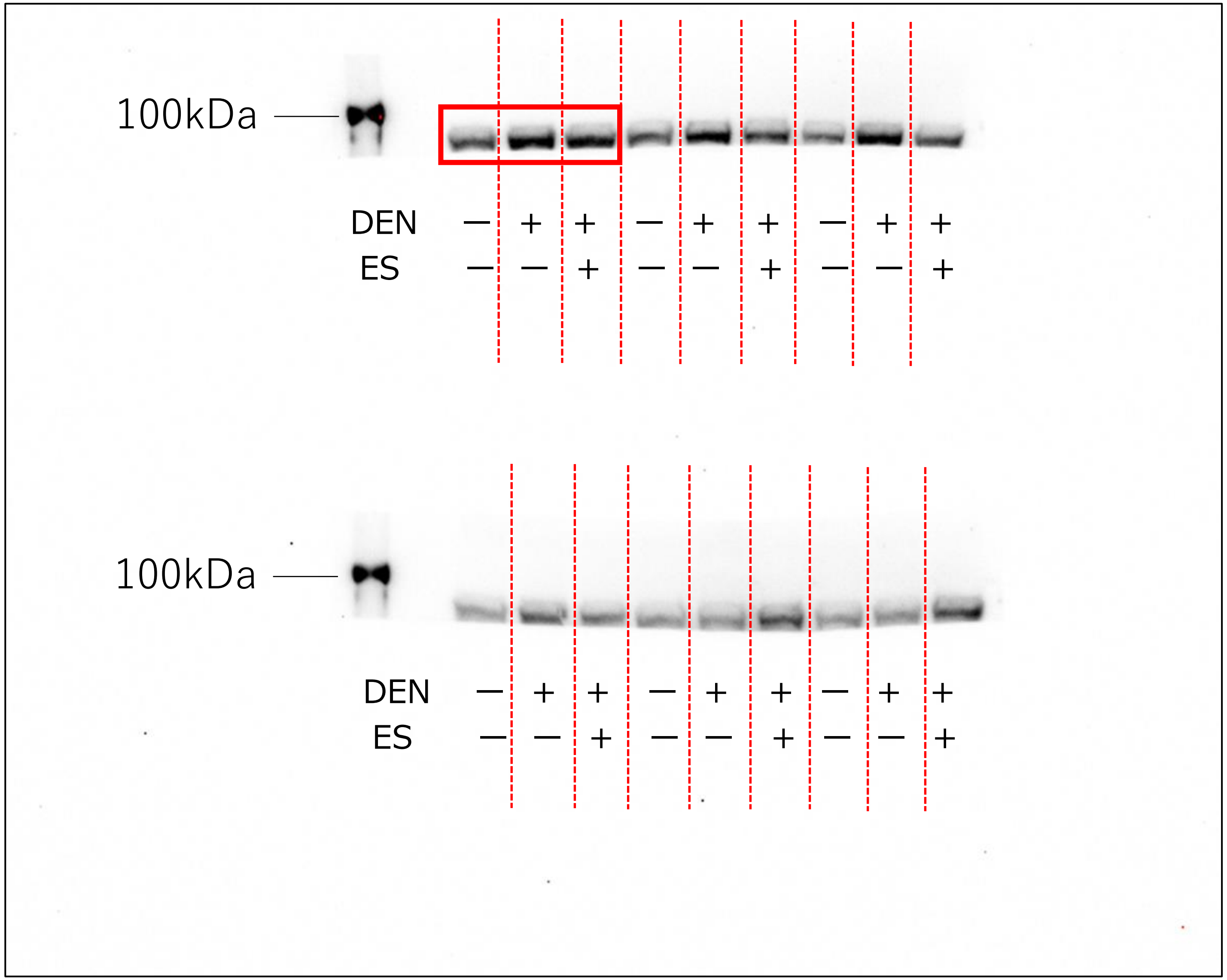

Supplementary Figure S6 b: Western blot membrane images of TIF-1a.

The membrane was cut at the position of the desired molecular weight and the antibody reaction was performed. 2 membranes were photographed simultaneously for analysis.

Red frame: used in the figure.

Tibialis anterior

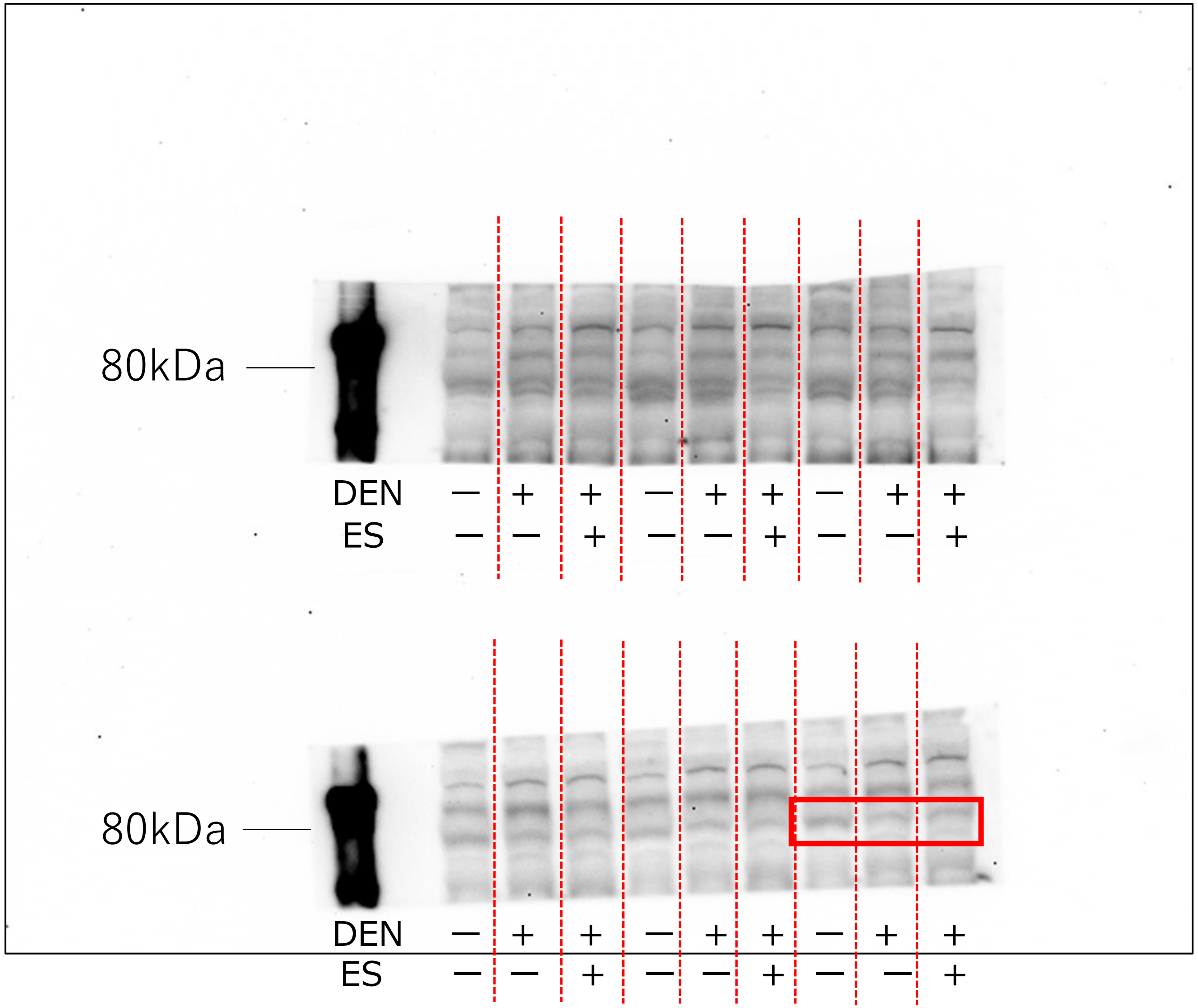

Supplementary Figure S6 b: Western blot membrane images of TIF-1a.

The membrane was cut at the position of the desired molecular weight and the antibody reaction was performed. 2 membranes were photographed simultaneously for analysis.

Red frame: used in the figure.

Gastrocnemius

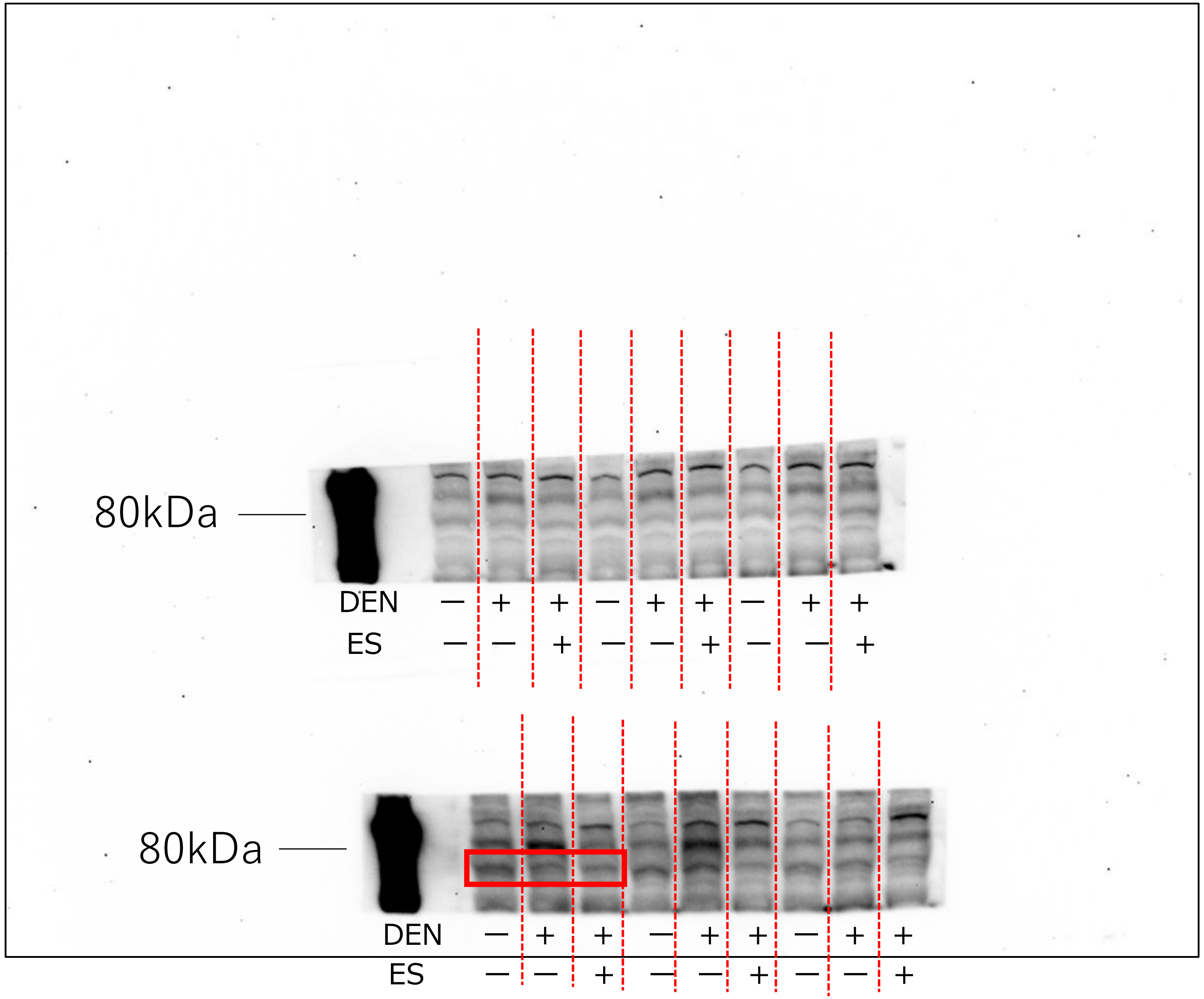

Supplementary Figure S6 c: Western blot membrane images of c-myc.

The membrane was cut at the position of the desired molecular weight and the antibody reaction was performed. 2 membranes were photographed simultaneously for analysis.

Red frame: used in the figure.

Tibialis anterior

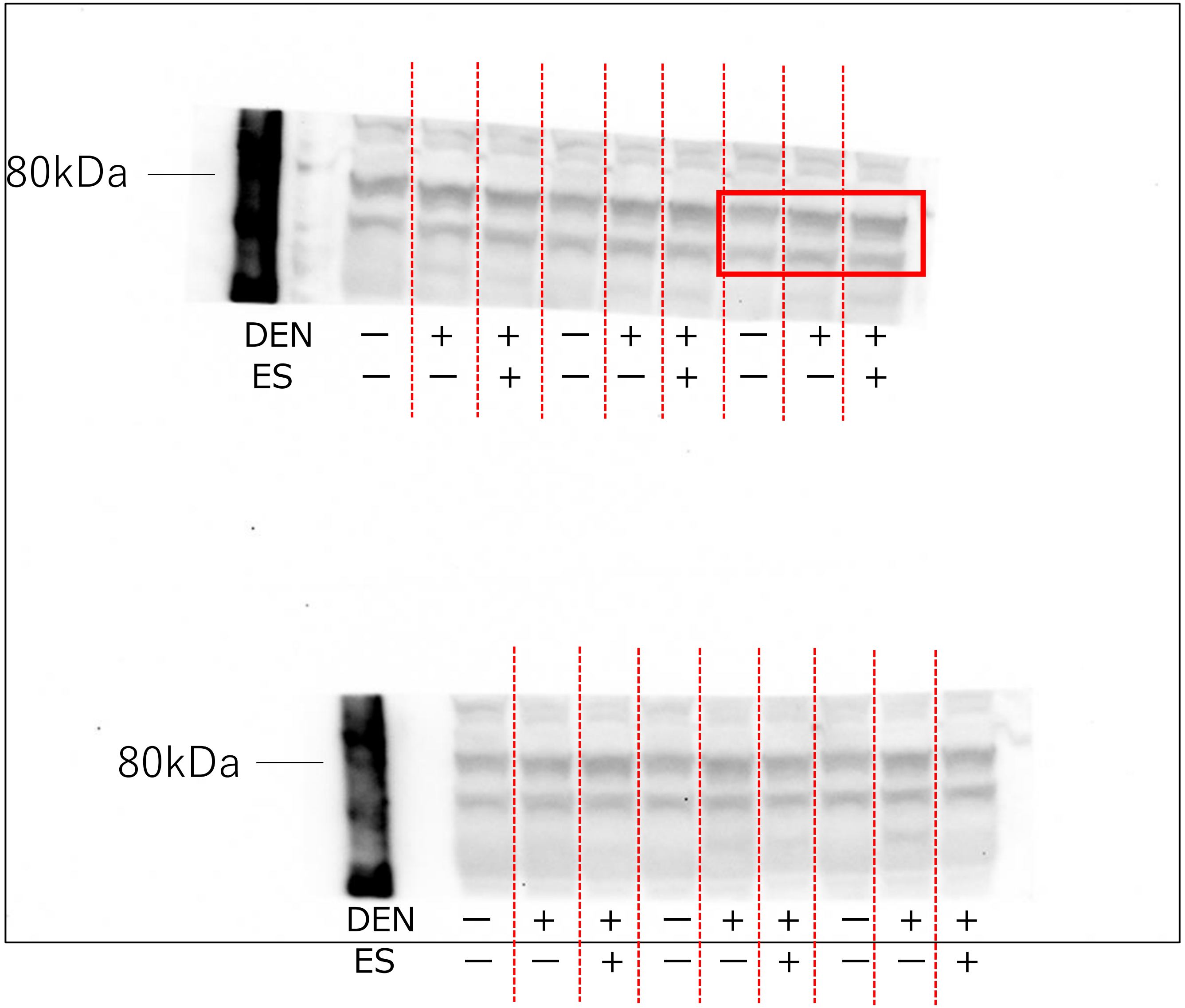

Supplementary Figure S6 c: Western blot membrane images of c-myc.

The membrane was cut at the position of the desired molecular weight and the antibody reaction was performed. 2 membranes were photographed simultaneously for analysis.

Red frame: used in the figure.

Gastrocnemius

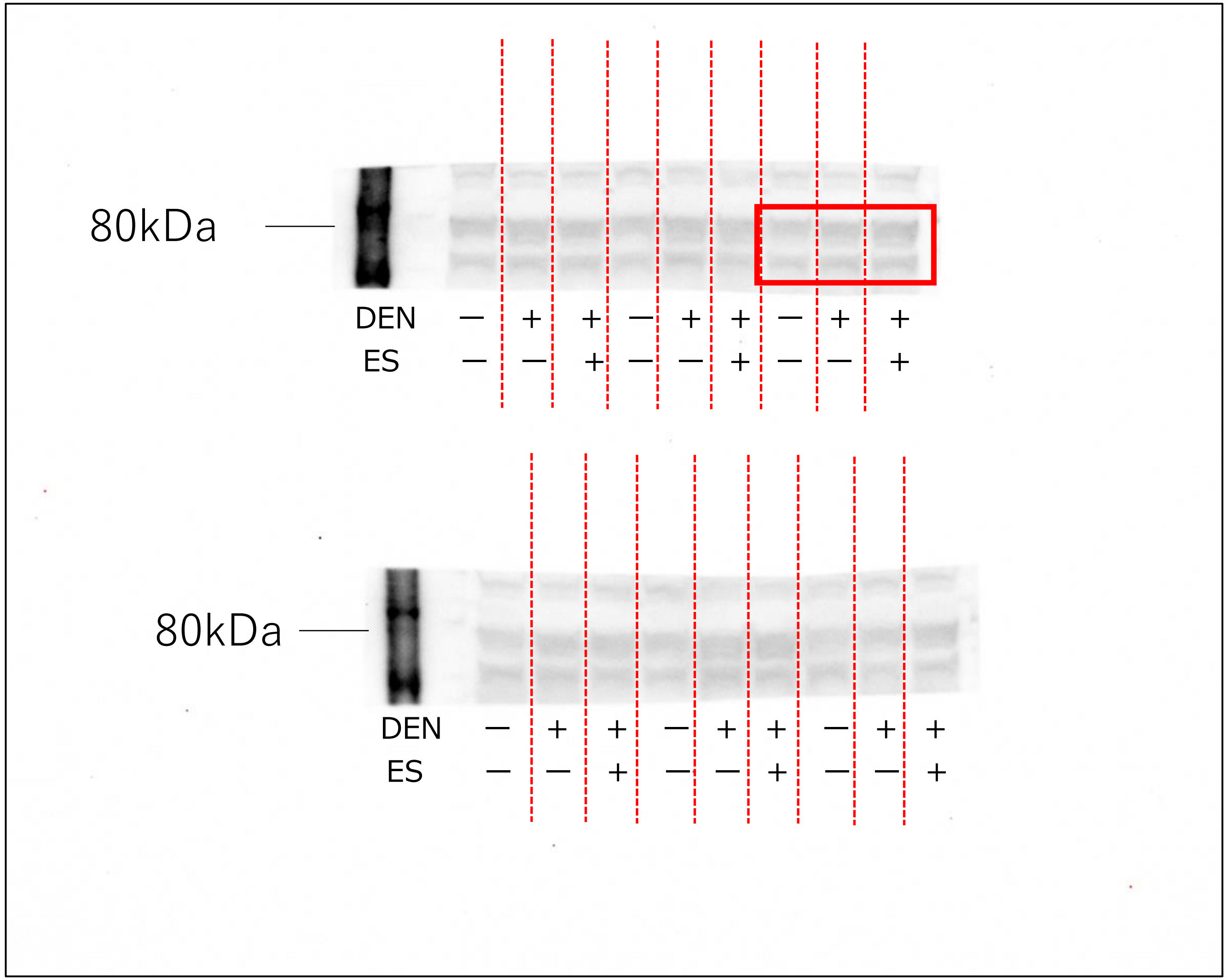

Supplement: Supplementary file 1 — Supplementary Information. [file 41598_2024_56382_MOESM1_ESM.pdf]
